# SMCFO: a novel cuttlefish optimization algorithm enhanced by simplex method for data clustering

**DOI:** 10.3389/frai.2025.1677059

**Published:** 2025-09-26

**Authors:** Kalpanarani K., Hannah Grace G.

**Affiliations:** Department of Mathematics, School of Advanced Sciences, Vellore Institute of Technology – Chennai, Chennai, Tamil Nadu, India

**Keywords:** clustering, cuttlefish optimization algorithm, Nelder-Mead simplex method, global search ability, metaheuristic optimization algorithm

## Abstract

**Introduction:**

In unsupervised learning, data clustering is essential. However, many current algorithms have issues like early convergence, inadequate local search capabilities, and trouble processing complicated or unbalanced input. Established methods like Kmeans are still widely used because of their ease of use; however, they struggle with non-spherical cluster shapes, which are sensitive to initialization, and suffer in highdimensional space. As a substitute, metaheuristic algorithms have surfaced as possible options, providing powerful global search ability. The Cuttlefish Optimization Algorithm (CFO) shows promise in clustering applications but suffers from premature convergence and poor local optimization capability.

**Methods:**

This paper introduces a new clustering method based on the Cuttlefish Optimization Algorithm (CFO), which improves upon the Nelder-Mead simplex method known as SMCFO. The method partitions the population into four subgroups with specific update strategies. One subgroup uses the Nelder-Mead method to improve the quality of solutions, while the others attempt to maintain exploration and exploitation equilibrium. This study compares the performance of the suggested SMCFO algorithm with four established clustering algorithms: CFO, PSO, SSO, and SMSHO. The evaluation used 14 datasets, which include two artificial datasets and 12 benchmark datasets sourced from the UCI Machine Learning Repository.

**Results and discussion:**

The proposed SMCFO algorithm consistently outperformed competing methods across all datasets, achieving higher clustering accuracy, faster convergence, and improved stability. The robustness of these outcomes was further confirmed through nonparametric statistical tests, which demonstrated that the performance improvements of SMCFO were statistically significant and not due to chance. The results confirm that the simplex-enhanced design boosts local exploitation and stabilizes convergence, which underlies SMCFO's superior performance compared to baseline methods.

## 1 Introduction

Data clustering is a fundamental unsupervised machine learning method that utilizes the inherent structures of the dataset to group related data points. Organizing data into clusters facilitates pattern recognition (Gupta and Kumar, 2024), anomaly detection (Ali et al., 2024), and efficient data summarization. Various domains widely use it, including image processing (Bhimavarapu et al., 2024), customer segmentation (Vijilesh et al., 2021), genetics (Wu et al., 2021), and network analysis (Li et al., 2023), and blockchain for transaction analysis and fraud detection (Yang et al., 2025).

Clustering is considered an NP-hard problem. It identifies the cluster center for each cluster to classify all the data reasonably. Despite its versatility, no single clustering algorithm is optimal for all clustering problems. The effectiveness of a clustering method depends on several factors, including data distribution, cluster density, shape, and noise levels. These elements are essential for optimizing clustering results.

### 1.1 Existing clustering methods and critical analysis

Traditional clustering approaches include:

- Partitional clustering, such as K-Means (Oti et al., 2021), minimizes the variation within each cluster to group data into k clusters. As long as the clusters remain unstable, points will be continuously allocated to the nearest centroid. Subsequently, the centroids will be updated by computing the mean of the points assigned to each cluster. Spherical and well-separated clusters operate well, but more complex configurations cause problems.
- Hierarchical clustering (Ran et al., 2023) involves dividing bigger clusters (divisive) or continually merging smaller clusters (agglomerative) to create a hierarchy of clusters. The number of clusters need not be known in advance, and a dendrogram is frequently used to display the results. Although it excels at capturing nested relationships, the benefit can become computationally costly when dealing with huge datasets.
- Density-based clustering finds clusters as densely populated areas of points with sparser regions in between. Algorithms such as DBSCAN (Bushra and Yi, 2021) can identify clusters of any shape and deal with noise, which makes them applicable to complex datasets. Their performance can be parameter-sensitive and may not be well-suited for dealing with mixed cluster densities.
- Model-based clustering (Gormley et al., 2023) techniques treat data as coming from a combination of probability distributions. They effectively capture complex cluster shapes and assign data points based on probabilities. Still, they require high computational resources and rely heavily on correct model assumptions and the predefined number of clusters.
- Grid-based clustering (Tareq et al., 2021) partitions the data space into a grid of cells and forms clusters based on the data density within each cell. This method is efficient for large datasets, but its performance depends on the grid size and can struggle with irregular cluster shapes.

While these methods provide practical frameworks, they often face challenges in high-dimensional, nonlinear, or noisy datasets. To overcome these, researchers have explored metaheuristic and bio-inspired optimization techniques for clustering.

#### 1.1.1 Metaheuristic and bio-inspired clustering approaches

Researchers employ Genetic Algorithm (GA)-based clustering to enhance adaptability and performance across domains. Lin et al. (2005) improved fitness evaluation through pre-computed distance look-ups and efficient cluster center selection. GA-based approaches for Wireless Sensor Networks (Batra and Kant, 2018) optimized cluster formation and energy efficiency. Trajectory-based clustering methods (Yang et al., 2020) and fuzzy TDA-based F-Mapper (Bui et al., 2020) enhanced clustering quality and robustness against noise.

Swarm intelligence techniques have become more popular. For instance, variations based on PSO (Rengasamy and Murugesan, 2021; Niu et al., 2017) enhance the accuracy and speed of convergence. SHO and its simplified variant, SMSHO, improve centroid precision and population diversity (Fausto et al., 2017; Zhao et al., 2021; Anand and Arora, 2020). Similarly, Social Spider Optimization (SSO) (Thalamala et al., 2019; Buvanesvari and Begum, 2020; Zhao et al., 2021) has effectively discovered communities and clustering text. Exploration and exploitation are well-balanced. Feature selection (Karunakaran et al., 2020), dimensionality reduction (Suganthi and Karunakaran, 2019), and image segmentation (Bhandari et al., 2019) have all seen successful applications of the Cuttlefish Optimization (CFO) algorithm (Kowalski et al., 2020). It encourages solution variety through the use of visibility and reflection methods.

Even with their promising results, earlier clustering and metaheuristic methods have limitations. Numerous CFO variations and associated bio-inspired algorithms heavily rely on random operators. This reliance could lead to early convergence and less stability in complex search spaces. Moreover, inefficient local exploitation sometimes leads to inaccurate centroid refinement, especially in high-dimensional or nonlinear datasets. These issues show that a better approach that balances improved local search with global exploration is required. The simplex enhancement in CFO suggested in this study is motivated by these problems.

The new SMCFO selectively incorporates simplex into Group I of the CFO population, unlike SMSHO and SMSSO, which use the simplex method as an extra operator during the restoration or communication stages. Only the refinement group improves with deterministic local search in this architecture, but Groups II-IV maintain their unique exploratory responsibilities. By combining the geometric changes of the simplex method for updating centroids with the reflection and visibility dynamics of the CFO, SMCFO offers a new approach. This selective integration is structurally different from earlier simplex-hybrid methods. It leads to more effective clustering and more reliable, unique solutions.

### 1.2 Motivation and contribution

The literature demonstrates that there are still several issues with current clustering algorithms. Many tend to converge slowly and produce imprecise results. They frequently require numerous iterations to achieve satisfactory performance. Furthermore, researchers often use small datasets to test algorithms, making generalizing results challenging. Handling high-dimensional data remains another significant hurdle. These limitations highlight the need for improved approaches, which is the central focus of this work.

A detailed analysis of the CFO's performance shows it has a limited global search ability, often leading to locally optimal solutions. Introducing the simplex method enhances the CFO algorithm, improving its ability to explore the search space and avoid local optima.

The key contributions of this paper are outlined below:

1. The Simplex method is incorporated into the CFO algorithm for the first time to improve its performance. This incorporation substitutes some conventional operations in the CFO with reflection, expansion, contraction, and shrinking operations to improve local search. The algorithm attains a more balanced global exploration and local exploitation by improving population diversity and candidate solution quality. Consequently, it enhances convergence rate, scalability, and stability and decreases computational complexity. This improvement makes the optimized algorithm more effective for centroid-based clustering, nonlinear data structures, and high-dimensional data scenarios.
2. The proposed algorithm is applied to solve data clustering problems, with its effectiveness evaluated through comprehensive experiments on 14 widely used classified datasets from the UCI repository. The experiments assess various aspects of the algorithm's performance, including (1) overall optimization capability, (2) convergence speed, (3) result variance, (4) quality of visual clustering, (5) diversity of generated solutions, (6) algorithmic stability, (7) computational runtime, (8) impact of population size, (9) effect of the number of iterations, (10) statistical significance based on nonparametric rank-sum tests, and (11) evaluation using standard performance metrics such as accuracy, F-measure, sensitivity, specificity, and Adjusted Rand Index (ARI).
3. This study compares the proposed algorithm with several established clustering algorithms, including PSO (Rengasamy and Murugesan, 2021), SSO (Thalamala et al., 2019), SMSHO (Zhou et al., 2017), and CFO (Eesa et al., 2013). It performs a thorough analysis and evaluation of these algorithms in the context of data clustering. The experimental results reveal that the proposed algorithm attains faster convergence, higher accuracy, and more excellent stability than the other methods.

The structure of this paper is as follows: Section 2 introduces the mathematical model for data clustering. Section 3 outlines the cuttlefish optimization algorithm. Section 4 provides a detailed discussion of a variant of the cuttlefish optimization algorithm based on the simplex method (SMCFO). Section 5 presents the simulation experiments related to data clustering and analyzes the results. Finally, Section 6 concludes the study and suggests directions for future work.

## 2 Mathematical framework for clustering analysis

Data clustering analysis aims to classify data based on their inherent properties. Each group consists of highly similar elements, while elements in different groups exhibit significant differences. The following expressions define the mathematical framework for clustering analysis (Ma et al., 2015).

### 2.1 Description of the data clustering problem

Consider a dataset represented as: $D=\left\{{\text{x}}_{1},{\text{x}}_{2},\ldots ,{\text{x}}_{N}\right\}$ where each data point **x**_*i*_ is an *M*-dimensional vector: **x**_*i*_ = (*x*_*i*1_, *x*_*i*2_, …, *x*_*iM*_) for *i* = 1, 2, …, *N*. The objective of clustering is to partition D into *K* disjoint clusters: $G=\left\{G_{1},G_{2},\ldots ,G_{K}\right\}$ such that the following conditions hold:

1. Each cluster is non-empty:
  (1) $$\begin{array}{l} G_{k}\neq \emptyset ,\;\forall k\in \left\{1,2,\ldots ,K\right\}. \end{array}$$
2. Clusters are mutually exclusive:
  (2) $$\begin{array}{l} G_{i}\cap G_{j}=\emptyset ,\;\forall i,j\in \left\{1,2,\ldots ,K\right\},\;i\neq j. \end{array}$$
3. The union of all clusters reconstructs the original dataset:
  (3) $$\begin{array}{l} \overset{K}{\underset{k=1}{⋃}}G_{k}=D. \end{array}$$

Each cluster *G*_*k*_ groups data points with high similarity based on a predefined metric, minimizing intra-cluster variations and maximizing inter-cluster differences.

### 2.2 Principles of clustering

In the clustering process, the dataset D is divided into *K* clusters, denoted as {*G*_1_, *G*_2_, …, *G*_*K*_}. Each cluster $G_{j}$ has a representative centroid **z**_*j*_, where *j* = 1, 2, …, *K*. The set of all cluster centroids represents: $Z=\left\{{\text{z}}_{1},{\text{z}}_{2},\ldots ,{\text{z}}_{K}\right\}.$ The goal of clustering is to find the optimal set of centroids Z that ensures a significant degree of resemblance between data points in the same cluster while maximizing the distinction between different clusters. To measure similarity, the Euclidean distance function computes the distance between a data point **x**_*i*_ and the cluster centroid **z**_*j*_ as follows:


(4) $$\begin{array}{l} d\left({\text{x}}_{i},{\text{z}}_{j}\right)=\sqrt{\overset{M}{\underset{p=1}{\sum }}{\left(x_{i,p}-z_{j,p}\right)}^{2}}, \end{array}$$

where *M* is the number of attributes in the dataset, *x*_*i, p*_ is the *p*^*th*^ attribute of the data point **x**_*i*_, and *z*_*j, p*_ is the *p*^*th*^ attribute of the cluster centroid **z**_*j*_. Each data point **x**_*i*_ is assigned to the cluster whose centroid is closest in Euclidean distance. Formally, the assignment is defined as: ${\text{x}}_{i}\in G_{q}\;\text{if}\;d\left({\text{x}}_{i},{\text{z}}_{q}\right)<d\left({\text{x}}_{i},{\text{z}}_{r}\right),\;\forall r\neq q.$ This clustering procedure maximizes intra-cluster similarity while inter-cluster variations remain significant.

### 2.3 Optimization function for clustering

The SMCFO algorithm is developed to enhance clustering performance by addressing the limitations of traditional methods. Consider a dataset D with *N* data points, where each data point **x**_*i*_ has *M* attributes. The objective is to partition D into *K* clusters, each represented by a centroid **z**_*k*_. The complete set of centroids is given by: $Z=\left\{{\text{z}}_{1},{\text{z}}_{2},\ldots ,{\text{z}}_{K}\right\}.$ The dimension of the final result is a *K*×*M* matrix, which helps to optimize the cluster centroids. In the SMCFO algorithm, each individual represents a clustering center vector corresponding to a potential solution. A well-formed clustering solution minimizes intra-cluster distances while ensuring distinct separation between clusters. The clustering process aims to minimize the within-cluster sum of squares (WCSS), expressed as


(5) $$\begin{array}{l} \underset{Z}{\min}f\left(D,Z\right)=\overset{K}{\underset{k=1}{\sum }}\underset{{\text{x}}_{i}\in G_{k}}{\sum }∥{\text{x}}_{i}-{\text{z}}_{k}{∥}^{2}. \end{array}$$

Here, D represents the dataset and Z is the clustering center vector. The SMCFO algorithm seeks to determine the optimal centroid set Z that minimizes this function, ensuring compact and well-separated clusters.

## 3 Cuttlefish optimization algorithm (CFO)

The CFO is a global optimization method inspired by the natural behavior of cuttlefish. This algorithm was introduced by Eesa et al. (2013). A distinctive characteristic of cuttlefish, which underpins this algorithm (Eesa et al., 2014), is their ability to exhibit individual behaviors that collectively resemble those of a larger group. The algorithm draws inspiration from the cuttlefish's remarkable ability to change color. This trait serves as camouflage against predators and a strategy for attracting mates during reproduction. This dynamic color change is produced by light reflecting off multiple layers of specialized skin cells, including chromatophores, leucophores, and iridophores, which generate various patterns and colors. The CFO leverages this biological phenomenon by incorporating two key processes: reflection and visibility. These mechanisms simulate how cuttlefish adjust their appearance in response to environmental stimuli, guiding the algorithm's search for new, optimal solutions.

The process of generating a new solution in the optimization task, guided by reflection and visibility, is illustrated in Equation 6.


(6) $$\begin{array}{l} X_{new}=reflection+visibility. \end{array}$$

The CFO algorithm divides individuals into four groups, each employing different mechanisms to generate new solutions. In computational intelligence, these mechanisms correspond to acquiring novel solutions:

**Group I**: Solutions are updated based on the current position.


(7) $$\begin{array}{lll} reflection & = & R\cdot X_{c}, \end{array}$$

(8) $$\begin{array}{lll} visibility & = & V\cdot \left(X_{best}-X_{c}\right). \end{array}$$

**Group II**: Solutions are influenced by current and best positions.


(9) $$\begin{array}{lll} reflection & = & R\cdot X_{best}, \end{array}$$

(10) $$\begin{array}{lll} \text{visibility} & = & V\cdot \left(X_{best}-X_{c}\right). \end{array}$$

**Group III**: Solutions are generated based on the best-known position.


(11) $$\begin{array}{lll} reflection & = & R\cdot X_{best}, \end{array}$$

(12) $$\begin{array}{lll} visibility & = & V\cdot \left(X_{best}-X_{avg}\right). \end{array}$$

**Group IV**: A random search is performed within the solution space.


(13) $$\begin{array}{lll} reflection & = & \xi , \end{array}$$

(14) $$\begin{array}{lll} visibility & = & 0. \end{array}$$

Here, *X*_*new*_ represents a candidate solution, *X*_*c*_ denotes the current solution, and *X*_*best*_ is the best solution found so far. The term ξ represents a randomly selected position in the search space. The parameters *R* and *V* are random values drawn from uniform distributions over the intervals [*r*_1_, *r*_2_] and [*v*_1_, *v*_2_], respectively. *X*_*avg*_ denotes the average of the best solutions discovered during optimization.

Initialization, grouping, solution updates, evaluation, and iteration are all steps in the structured optimization process used by the CFO until a stopping criterion is satisfied. After initializing the population randomly, the algorithm divides it into four groups, each employing distinct reflection and visibility mechanisms to refine candidate solutions. The grouping strategy balances exploration and exploitation: Group I intensifies the search around the best solution, Groups II and III refine local searches for improved convergence, and Group IV introduces randomness to enhance diversity and prevent premature convergence. The efficiency of CFO depends on four key parameters (*r*_1_, *r*_2_, *v*_1_, *v*_2_), which regulate the balance between exploration and exploitation. Adjusting these parameters enhances the algorithm's capability to find optimal solutions.

## 4 A novel cuttlefish optimization algorithm enhanced by Nelder-Mead Simplex (SMCFO)

There are numerous applications for the CFO algorithm. Nevertheless, it has certain drawbacks, such as a tendency to become trapped in local optima, sensitivity to parameter settings, inefficient convergence, and high computing cost. To overcome these problems, the CFO of Group I has incorporated the Nelder-Mead Simplex approach instead of the conventional reflection and visibility procedures. The algorithm can converge more quickly and detect cluster centroids more precisely, which improves local search by dynamically modifying the search space. The Simplex method also helps prevent premature convergence and improves the CFO's ability to explore complex solution landscapes without relying on gradient information. By properly balancing the roles of exploration and exploitation, the improved CFO achieves better clustering performance with excellent stability and efficiency.

### 4.1 Justification of group selection for simplex integration

Incorporating the Nelder-Mead simplex method into Group I, instead of other CFO groups, was backed by theory and evidence. Each group in the CFO algorithm has a specific role: Group I focuses on refining, Group II and III emphasize guided exploration and focused exploitation. At the same time, Group IV maintains diversity through randomization. The Nelder-Mead simplex method is a local search technique that needs strong candidate solutions to work well, so Group I fits its exploitative nature. On the other hand, adding Nelder-Mead to Groups II-IV either conflicted with their exploratory goals, reduced exploration efficiency, or showed no meaningful performance improvement.

Additionally, comparative tests were conducted to validate this rationale by integrating simplex across different groups. The results indicated that adding it to Group I produced the most reliable gains in convergence rate, stability, and clustering accuracy.

Therefore, both the CFO's structural design and the experimental findings support restricting Nelder-Mead simplex to Group I, where it enhances local exploitation without undermining global search capability. The remaining groups continue to fulfill their original roles, ensuring that the proposed SMCFO maintains a robust balance between exploration and exploitation.

### 4.2 Local refinement using Nelder-Mead simplex method

**Figure 1:**
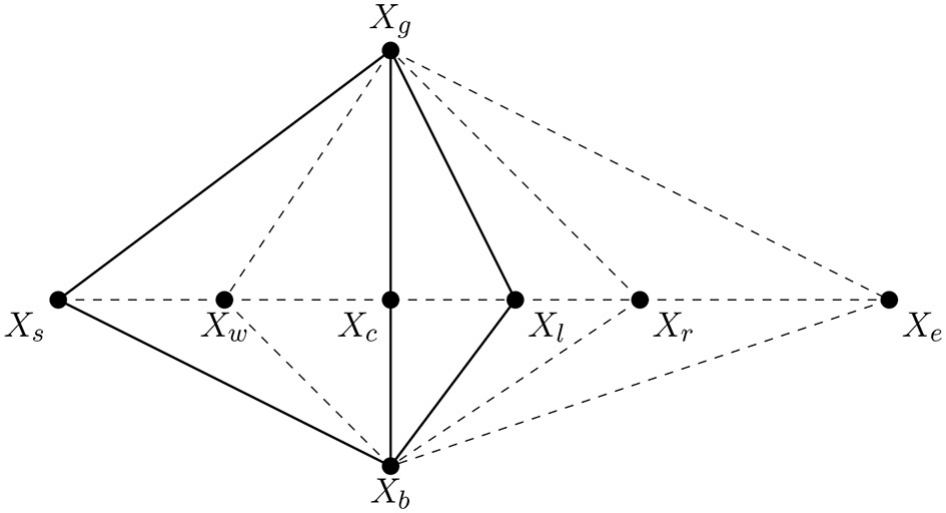
Diagrammatic overview of the method.

The proposed Simplex-Enhanced Cuttlefish Optimization (SMCFO) integrates the Nelder-Mead simplex method into Group I of the population. Individuals in this group are refined locally by the simplex method, rather than depending only on the original cuttlefish update rules. This hybridization improves local exploitation by adjusting centroid positions through geometric transformations like expansion, contraction, and reflection. As a result, SMCFO not only preserves global search through the remaining groups but also introduces dynamic local improvement to boost clustering precision and solution stability. This paper references the simplex method from Nelder and Mead (1965) and depicts a diagrammatic overview of the method in Figure 1. The algorithm steps are as follows:

**Step 1:** Evaluate the objective function for all individuals in the population and identify the optimal solution *x*_*g*_ and the suboptimal solution *x*_*b*_. Let *x*_*s*_ be an individual targeted by a predator. The objective function values of *x*_*g*_, *x*_*b*_, and *x*_*s*_ are denoted as *f*(*x*_*g*_), *f*(*x*_*b*_), and *f*(*x*_*s*_), respectively.
**Step 2:** Compute the central position *x*_*c*_ of *x*_*g*_ and *x*_*b*_ using the following formula:
  (15) $$\begin{array}{l} x_{c}=\frac{x_{g}+x_{b}}{2} \end{array}$$
**Step 3:** Perform the reflection operation using the formula:
  (16) $$\begin{array}{l} x_{r}=x_{c}+\alpha \left(x_{c}-x_{s}\right) \end{array}$$
  where *x*_*r*_ represents the reflection point of *x*_*s*_, and α is the reflection coefficient, typically set to 1.
**Step 4:** Evaluate the objective function values of *x*_*r*_ and *x*_*g*_. If *f*(*x*_*r*_) < *f*(*x*_*g*_), apply the expansion operation as follows:
  (17) $$\begin{array}{l} x_{e}=x_{c}+\gamma \left(x_{r}-x_{c}\right) \end{array}$$
  where γ is the expansion coefficient, typically set to 2. Next, compare *f*(*x*_*e*_) and *f*(*x*_*g*_). If *f*(*x*_*e*_) < *f*(*x*_*g*_), update *x*_*s*_ with *x*_*e*_; otherwise, replace *x*_*s*_ with *x*_*r*_.
**Step 5:** Compare the objective function values of *x*_*r*_ and *x*_*s*_. If *f*(*x*_*s*_) < *f*(*x*_*r*_), execute the contraction operation using:
  (18) $$\begin{array}{l} x_{t}=x_{c}+\beta \left(x_{s}-x_{c}\right) \end{array}$$
  where β represents the contraction coefficient, set to 0.5. Then, compare *f*(*x*_*t*_) and *f*(*x*_*s*_). If *f*(*x*_*t*_) < *f*(*x*_*s*_), substitute *x*_*s*_ with *x*_*t*_; otherwise, replace *x*_*s*_ with *x*_*r*_.
**Step 6:** If *f*(*x*_*g*_) < *f*(*x*_*r*_) < *f*(*x*_*s*_), apply the shrinking operation as follows:
  (19) $$\begin{array}{l} x_{w}=x_{c}-\delta \left(x_{s}-x_{c}\right) \end{array}$$
  where δ is the shrinking coefficient, typically set to 0.5 (Wang et al., 2016). This parameter is selected because it balances exploration and exploitation so that there can be stable convergence without premature stagnation. A coefficient of 0.5 ensures the gradual reduction in the search space with a bias toward refining solutions incrementally. Then, compare *f*(*x*_*w*_) and *f*(*x*_*s*_). If *f*(*x*_*w*_) < *f*(*x*_*s*_), update *x*_*s*_ with *x*_*w*_; otherwise, replace *x*_*s*_ with *x*_*r*_.

Group I improves local exploitation of promising candidate solutions by including the Nelder-Mead simplex approach. The improved individuals will enhance clustering accuracy and solution stability without sacrificing global exploration, based on our simplex operations analysis and the design of the SMCFO method. The simplex-enhanced architecture ensures effective convergence toward high-quality cluster centroids by balancing intensive local search and broad global exploration. The following section presents the complete SMCFO algorithm in flowchart and pseudocode form.

### 4.3 Pseudocode and flowchart of the novel SMCFO algorithm

**Figure 2:**
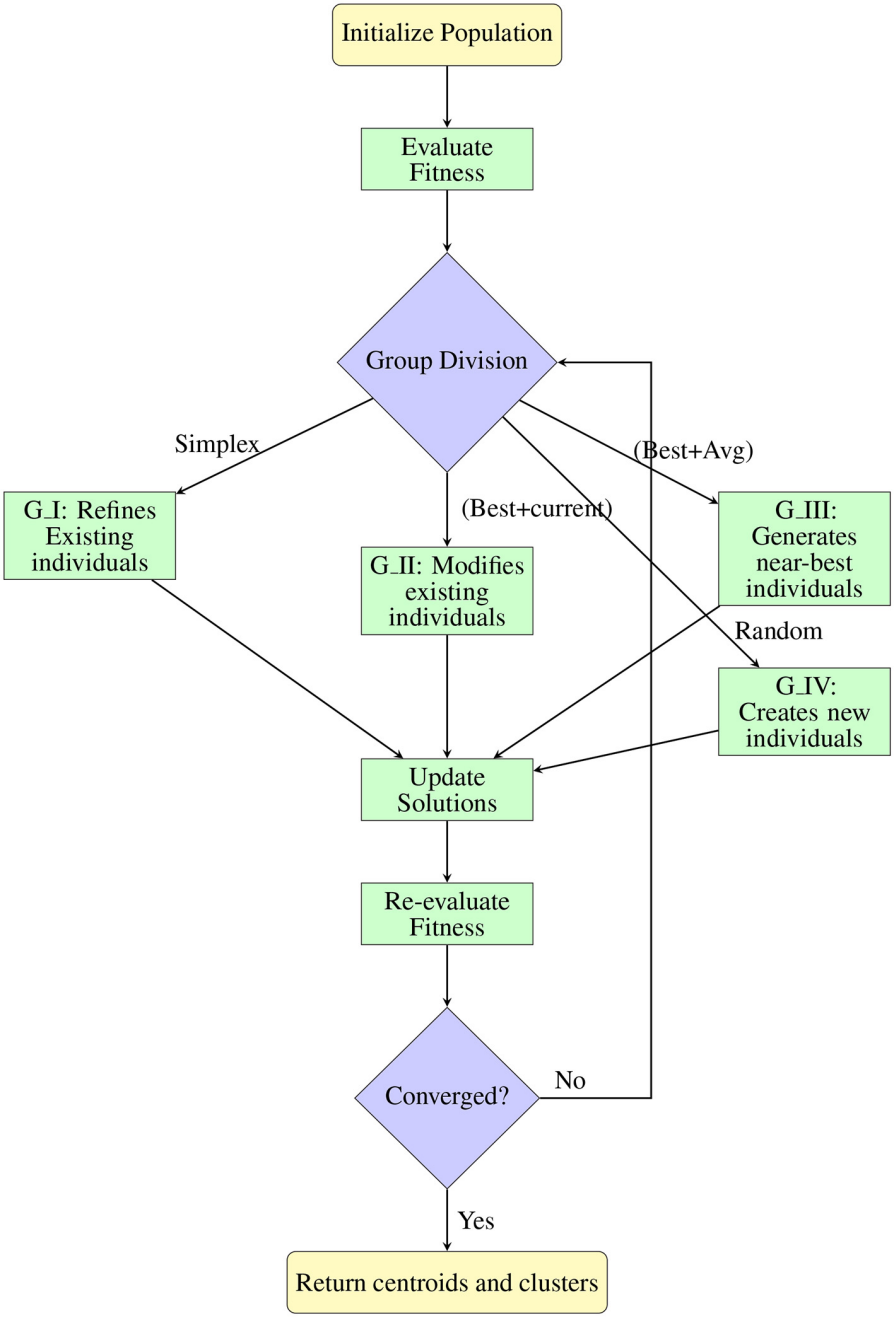
Flowchart of SMCFO algorithm process.

The pseudocode presented in Algorithm 1 outlines the step-by-step process of the Cuttlefish Optimization Algorithm enhanced by Nelder-Mead simplex method (SMCFO) for data clustering. The flowchart in Figure 2 illustrates the step-by-step procedure of the SMCFO for clustering. It shows how the population is initialized, divided into strategic groups, updated using CFO and simplex operations, and iteratively refined until convergence.

**Algorithm 1:** Pseudocode of novel SMCFO for data clustering.

**Initialize** Set up and structure the necessary data components for the algorithm
Generate a random initial population representing the positions of the swarm of cuttlefish.
Evaluate the fitness of each individual based on its position.
Identify the best global solution.
Divide the population into four subgroups: *G*_*I*_, *G*_*II*_, *G*_*III*_, and *G*_*IV*_.
**while** stopping criteria are not met **do**
Compute the average position of the best global solution *X*_avg_.
**for** each individual in *G*_*I*_ (Simplex Method applied) **do**
Apply the Nelder-Mead Simplex Method for local search refinement using Equations 15–19.
Update positions based on Simplex transformations.
Update the current position if a better solution is found.
**end for**
**for** each individual in *G*_*II*_ **do**
Compute reflection using Equation 9.
Compute visibility using Equation 10.
Generate a new position based on Equation 6.
Update the current position if a better solution is found.
**end for**
**for** each individual in *G*_*III*_ **do**
Compute reflection using Equation 11.
Compute visibility using Equation 12.
Generate a new position based on Equation 6.
Update the current position if a better solution is found.
**end for**
**for** each individual in *G*_*IV*_ **do**
Generate a new position based on Equations 6, 10–11.
Update the current position if a better solution is found.
**end for**
Check boundary constraints for newly generated positions.
Evaluate the fitness of each individual.
Update the best global solution if a better fitness value is obtained.
**end while**
**Return:** The optimal fitness value, corresponding cluster centroids, and assigned cluster labels of the dataset

## 5 Experimental results and discussions

A comprehensive series of tests is conducted to validate the proposed SMCFO's effectiveness in clustering tasks. The experimental environment is configured to ensure consistent evaluation conditions across diverse datasets—the following subsections present details of the experimental setup, datasets, and comparative methodologies.

### 5.1 Experimental environment

All the algorithms are executed in MATLAB online. The experiments are conducted on a laptop with an AMD Ryzen 7 7735U processor, Radeon Graphics (2.70 GHz), and 16 GB of memory.

### 5.2 Comparative analysis of algorithms

The proposed SMCFO algorithm significantly contributes to the study of data clustering analysis. The evaluation analyzes performance using 14 benchmark datasets, which include two synthetic datasets and 12 real-world datasets from the UCI Machine Learning Repository (Merz, 1998). Table 1 details the number of data points, features, and clusters for each dataset. All comparison algorithms will use the randomly selected parameters for the art1 and art2 datasets (Niknam and Amiri, 2010). Since each dataset has unique characteristics, no algorithm consistently achieves the best results in all experiments. Consequently, the experimental outcomes genuinely evaluate the proposed algorithm's performance.

**Table 1:** Details of the clustering datasets.

**Dataset**	**Data points**	**Features**	**Cluster**
art1	250	3	5
art2	600	2	4
iris	150	4	3
breastcancer	569	30	2
tae	151	5	3
heartstatlog	270	13	2
thy	215	5	3
haberman	306	3	2
cmc	1,473	10	3
glass	214	9	7
vehicle	946	18	4
vertebral	310	6	2
sonar	208	60	2
ecoli	336	7	8

To assess the effectiveness of the SMCFO algorithm in solving data clustering problems, we compare it with four other optimization algorithms: PSO Alswaitti et al., (2018), SSO (Cuevas et al., 2013; Cuevas and Cienfuegos, 2014), SMSHO (Fausto et al., 2017), and CFO (Kowalski et al., 2020). The parameters for these comparison algorithms are set as follows, based on prior studies and their ability to balance exploration and exploitation, enhance convergence behavior, and maintain diversity during optimization:

1. **PSO**: The weight factor is set to ω = 0.7298 with acceleration coefficients *c*_1_ = *c*_2_ = 1.4962, which is based on (Alswaitti et al. 2018). These values are widely adopted to achieve a good trade-off between exploration and exploitation, improving convergence and solution quality.
2. **SSO**: Parameters α, β, δ,*r*_*m*_ and *PF* are randomly selected within the interval [0, 1], as determined to be the optimal parameter set based on Cuevas et al. (2013) and Cuevas and Cienfuegos (2014). Randomization helps maintain population diversity and allows adaptive behavior across different problem instances.
3. **SMSHO**: Parameters α, β, γ, δ and ρ are randomly chosen within the range [0, 1], as referenced in Fausto et al. (2017). This stochastic approach increases global search ability and helps avoid local optima by enabling diverse solution exploration.
4. **CFO**: *r*_1_ = −0.5, *r*_2_ = 1.0, *v*_1_ = −2.0, *v*_2_ = 2.0, which is based on Kowalski et al. (2020). These parameters govern attraction-repulsion dynamics and have been empirically validated to facilitate effective search space exploration.

These experiments will compare the algorithms based on optimization performance, clustering effectiveness, solution diversity, stability, execution time, the influence of population size, the impact of iteration count, and the results of Wilcoxon's rank-sum test.

### 5.3 Comparison of algorithms based on optimization performance

Each experiment is independently repeated 30 times for each algorithm to minimize the impact of randomness in the comparison algorithms. Each run has a maximum of 200 iterations, with a population size 52 for all algorithms. The evaluation metrics include the best value, worst value, mean value, and standard deviation. Tables 2–16 present the experiment's findings, with the best-performing results indicated in bold. “Rank” denotes a thorough rating, usually based on the algorithm's optimization performance and obtained from the mean value.

**Table 2:** Comparative analysis of algorithms on art1.

**Algorithm**	**Best**	**Worst**	**Mean**	**SD**	**Rank**
PSO	**33.8521**	**95.3669**	**69.8576**	**0.0000**	**1**
SSO	74.1193	1,723.1142	285.7072	154.7319	3
SMSHO	155.6599	1,714.0961	515.5672	223.1702	5
CFO	117.0561	1,145.0481	360.0097	167.3484	4
SMCFO	95.7152	762.8250	100.5669	31.1964	2

Bold values indicate the best performance achieved for each metric among all compared methods.

**Table 3:** Comparative analysis of algorithms on art2.

**Algorithm**	**Best**	**Worst**	**Mean**	**SD**	**Rank**
PSO	**79.2966**	**190.2388**	**87.5799**	**0.0000**	**1**
SSO	86.0661	3,690.4179	452.8317	272.8619	4
SMSHO	117.0561	1,145.0481	360.0097	167.3484	3
CFO	161.4016	2,455.2082	561.6793	267.3990	5
SMCFO	186.7356	1,114.1836	223.3517	42.9580	2

Bold values indicate the best performance achieved for each metric among all compared methods.

**Table 4:** Comparative analysis of algorithms on iris.

**Algorithm**	**Best**	**Worst**	**Mean**	**SD**	**Rank**
PSO	78.9828	383.6866	102.8874	22.1032	2
SSO	140.5039	43,270.1360	1,764.5459	2,006.5924	5
SMSHO	78.8514	681.3706	153.3178	148.0128	3
CFO	155.6599	1,714.0961	515.5672	223.1702	4
SMCFO	**78.6346**	**156.6882**	**86.1619**	**20.2851**	**1**

Bold values indicate the best performance achieved for each metric among all compared methods.

**Table 5:** Comparative analysis of algorithms on breastcancer.

**Algorithm**	**Best**	**Worst**	**Mean**	**SD**	**Rank**
PSO	27,719.2213	61,961.8269	44,288.6832	0.0144	3
SSO	92,300.1836	2,703,176.1818	341,368.1412	141,670.5397	5
SMSHO	17,040.0000	17,040.0055	17,040.0002	7,403.4635	2
CFO	19,055.8592	286,259.9226	145,548.5798	40,091.9523	4
SMCFO	**12,346.6231**	**14,336.1204**	**14,270.620**	**0.0010**	**1**

Bold values indicate the best performance achieved for each metric among all compared methods.

**Table 6:** Comparative analysis of algorithms on tae.

**Algorithm**	**Best**	**Worst**	**Mean**	**SD**	**Rank**
PSO	461.2022	586.9884	505.4647	0.0008	3
SSO	501.2750	6,545.6577	1,059.1250	517.5986	5
SMSHO	**449.6920**	**570.3536**	**459.5600**	23.3462	**1**
CFO	598.7145	2,196.7815	919.4560	226.9308	4
SMCFO	460.7516	804.4755	493.6281	**15.4547**	2

Bold values indicate the best performance achieved for each metric among all compared methods.

**Table 7:** Comparative analysis of algorithms on heartstatlog.

**Algorithm**	**Best**	**Worst**	**Mean**	**SD**	**Rank**
PSO	3,210.0573	3,853.7981	3,499.9900	0.0018	3
SSO	4,074.4698	250,926.5770	21,650.6378	14,207.0077	5
SMSHO	**2,905.0868**	**3,497.0000**	**3,112.9941**	281.5282	**1**
CFO	3,378.3706	16,962.3370	7,584.6943	2,131.2829	4
SMCFO	3,119.3496	7,436.9606	3,201.8327	**238.0898**	2

Bold values indicate the best performance achieved for each metric among all compared methods.

**Table 8:** Comparative analysis of algorithms on thy.

**Algorithm**	**Best**	**Worst**	**Mean**	**SD**	**Rank**
PSO	475.0458	1,009.2961	761.1499	159.1832	3
SSO	772.5646	66,670.2281	4,352.6209	4,936.6877	5
SMSHO	**460.1975**	**756.8924**	**557.5196**	**115.3783**	**1**
CFO	938.9908	16,774.4383	3,901.8475	2,418.3636	4
SMCFO	669.7603	5,144.3083	721.2114	155.1949	2

Bold values indicate the best performance achieved for each metric among all compared methods.

**Table 9:** Comparative analysis of algorithms on haberman.

**Algorithm**	**Best**	**Worst**	**Mean**	**SD**	**Rank**
PSO	682.1756	719.3374	696.5331	**0.0000**	2
SSO	685.6081	24,970.0202	1,455.7101	1,756.6960	4
SMSHO	**682.1756**	**702.4383**	**686.9036**	8.7167	**1**
CFO	715.3827	11,736.1278	1,833.7641	1,341.1546	5
SMCFO	704.6310	1,574.9831	706.8229	23.5046	3

Bold values indicate the best performance achieved for each metric among all compared methods.

**Table 10:** Comparative analysis of algorithms on cmc.

**Algorithm**	**Best**	**Worst**	**Mean**	**SD**	**Rank**
PSO	10,178.1010	13,124.8768	11,600.2101	**0.0001**	3
SSO	12,947.5107	530,547.6987	49,936.6772	33,291.3189	5
SMSHO	**9,218.0206**	**11,322.6648**	**9,375.1683**	458.5580	**1**
CFO	12,021.3228	59,460.6542	23,606.7329	6,683.3060	4
SMCFO	9,867.2396	29,593.4094	10,224.7722	1,010.2569	2

**Table 11:** Comparative analysis of algorithms on glass.

**Algorithm**	**Best**	**Worst**	**Mean**	**SD**	**Rank**
PSO	1,559.1081	2,125.0225	1,745.3408	**0.0002**	3
SSO	2,110.1945	86,929.9417	12,884.2494	9,985.8961	5
SMSHO	**699.5876**	**1,421.2594**	**814.1782**	123.8344	**1**
CFO	1,664.1694	17,377.0621	6,540.1074	2,407.9114	4
SMCFO	997.7934	9,703.9430	1,203.9196	449.3489	2

Bold values indicate the best performance achieved for each metric among all compared methods.

**Table 12:** Comparative analysis of algorithms on vehicle.

**Algorithm**	**Best**	**Worst**	**Mean**	**SD**	**Rank**
PSO	13,894.2022	19,251.7790	16,789.8994	2,403.1634	3
SSO	37,486.7806	863,641.1042	184,219.0311	95,164.2036	5
SMSHO	8,287.4207	58,875.5762	14,222.7502	1,311.9655	2
CFO	14,612.3397	137,149.0716	53,779.8300	18,552.0968	4
SMCFO	**8,204.9647**	**15,210.1916**	**10,401.9627**	**0.3796**	**1**

Bold values indicate the best performance achieved for each metric among all compared methods.

**Table 13:** Comparative analysis of algorithms on vertebral.

**Algorithm**	**Best**	**Worst**	**Mean**	**SD**	**Rank**
PSO	1,087.5728	1,854.0548	1,377.5236	**0.0004**	2
SSO	1,798.0767	180,020.2325	12,638.3085	13,782.7860	5
SMSHO	**926.7104**	**1,854.0000**	**1,226.0398**	326.5232	**1**
CFO	1,630.7437	25,462.4495	6,055.7319	3,466.7428	4
SMCFO	1,206.5866	7,590.7959	1,450.6611	269.8318	3

Bold values indicate the best performance achieved for each metric among all compared methods.

**Table 14:** Comparative analysis of algorithms on sonar.

**Algorithm**	**Best**	**Worst**	**Mean**	**SD**	**Rank**
PSO	**410.1591**	**515.2461**	**466.0496**	**0.0000**	**1**
SSO	1,049.6481	6,335.2853	2,418.7901	515.2234	2
SMSHO	12,420.0000	12,432.2136	12,420.4071	2.2299	4
CFO	13,359.0858	84,317.0201	51,921.3700	10,102.2940	5
SMCFO	10,937.9891	54,500.3407	11,209.1078	2,678.6782	3

Bold values indicate the best performance achieved for each metric among all compared methods.

#### 5.3.1 Analyzing the effectiveness of optimization algorithms

**Figure 3:**
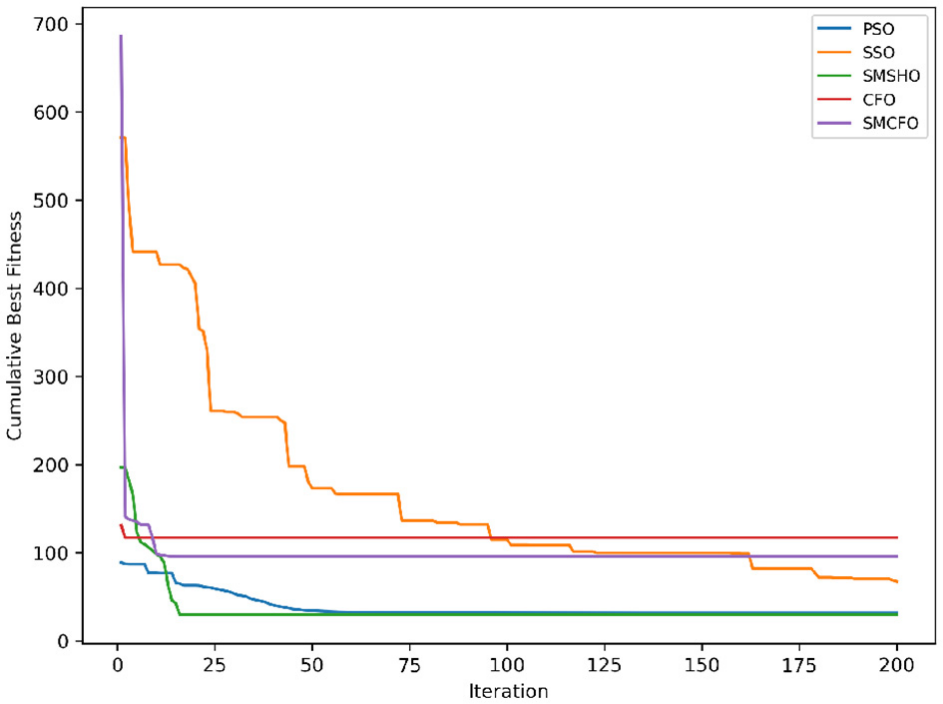
Evolutionary convergence trends for art1.

**Figure 4:**
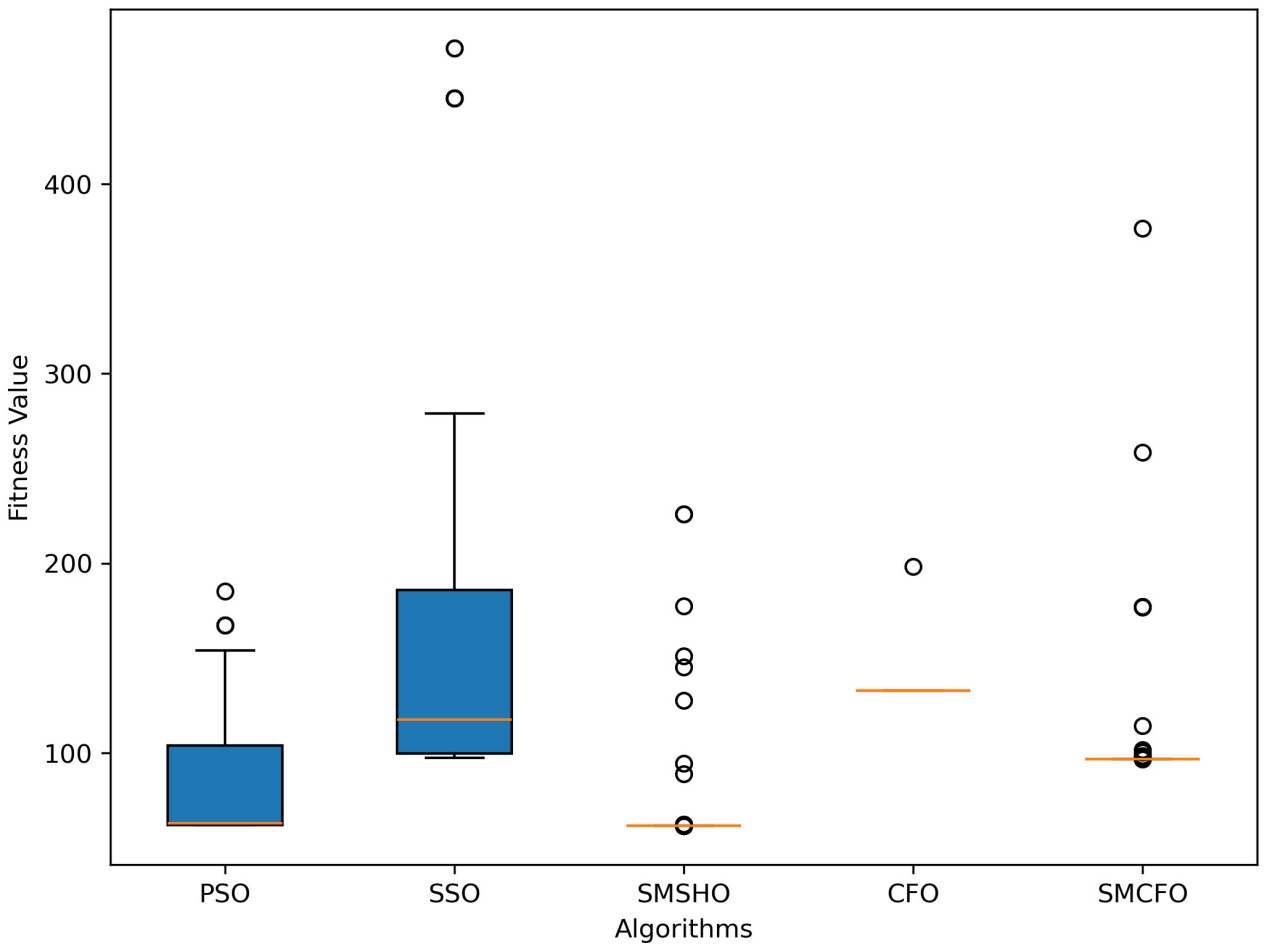
Variance analysis of optimization methods on art1.

**Figure 5:**
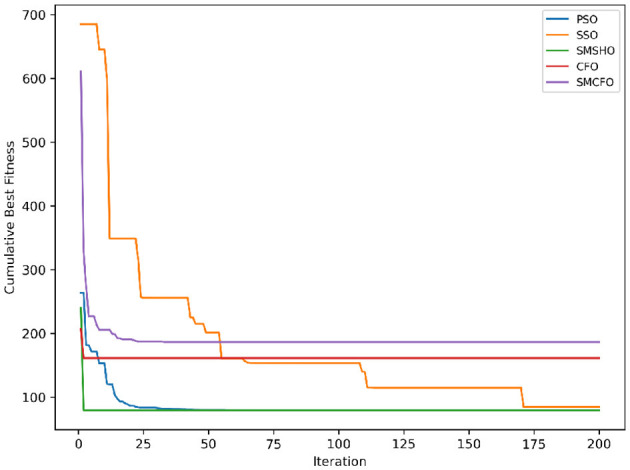
Evolutionary convergence trends for art2.

**Figure 6:**
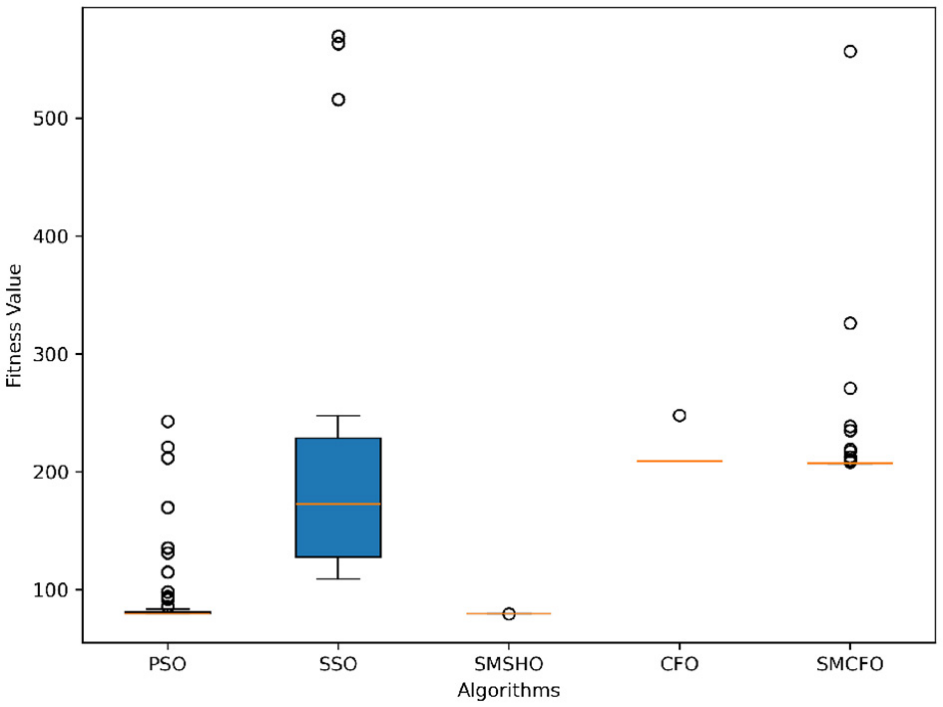
Variance analysis of optimization methods on art2.

**Figure 7:**
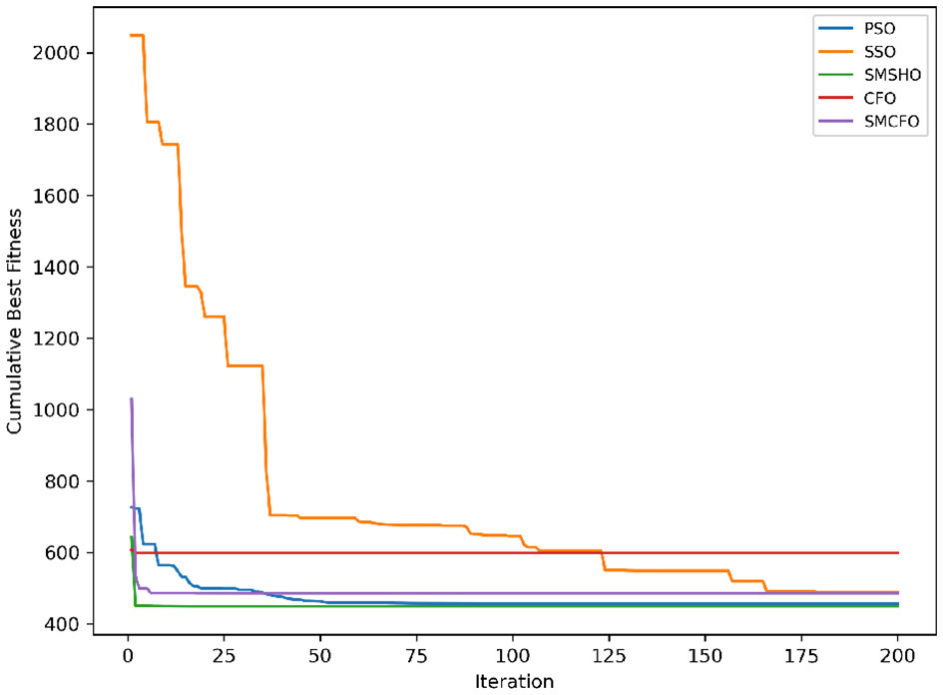
Evolutionary convergence trends for tae.

**Figure 8:**
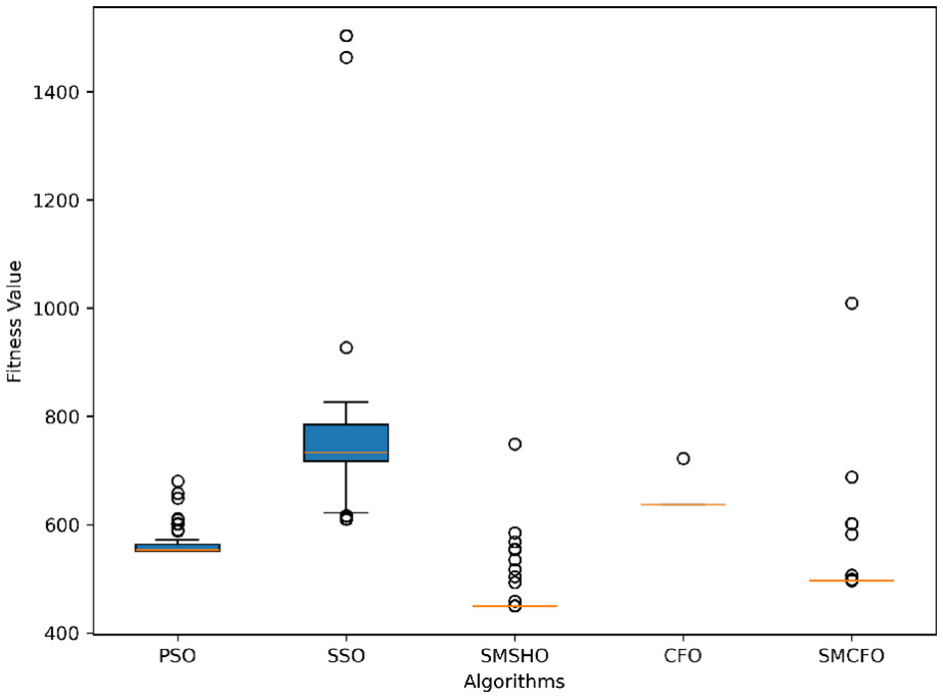
Variance analysis of optimization methods on tae.

**Figure 9:**
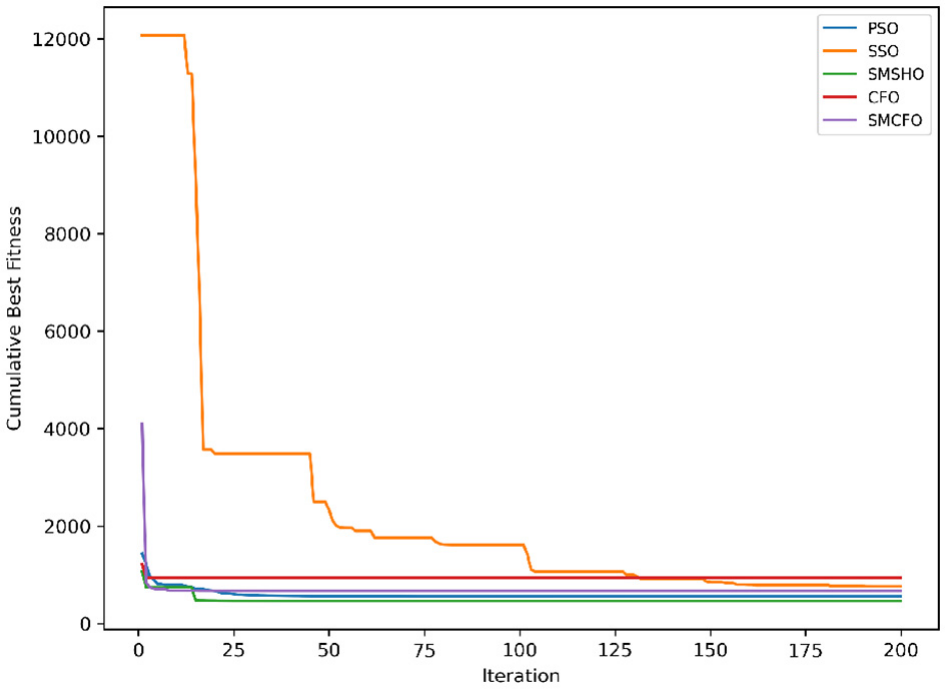
Evolutionary convergence trends for thy.

**Figure 10:**
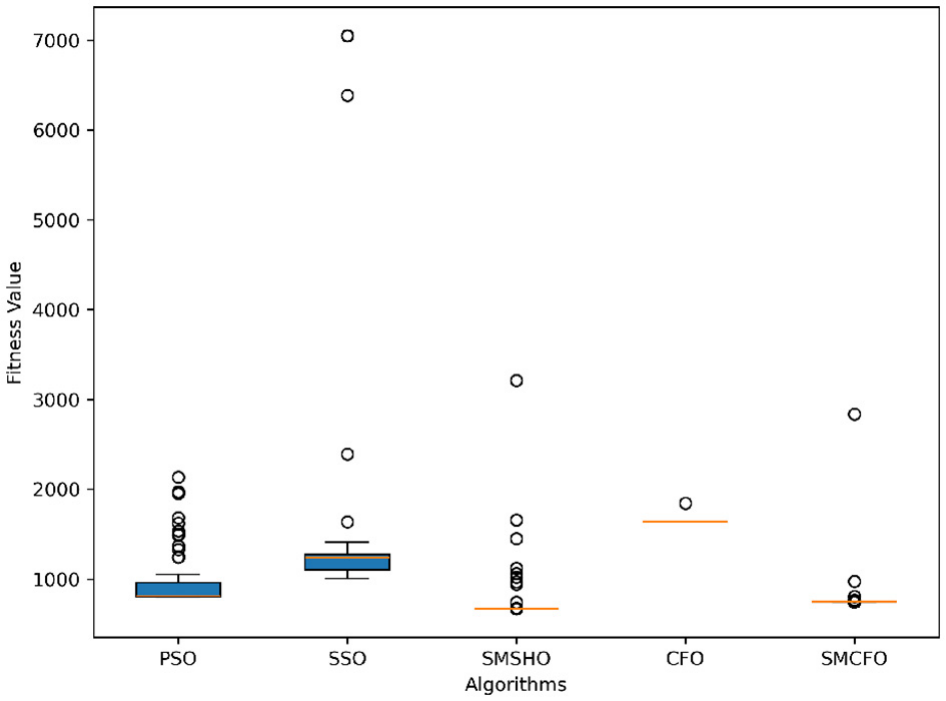
Variance analysis of optimization methods on thy.

**Figure 11:**
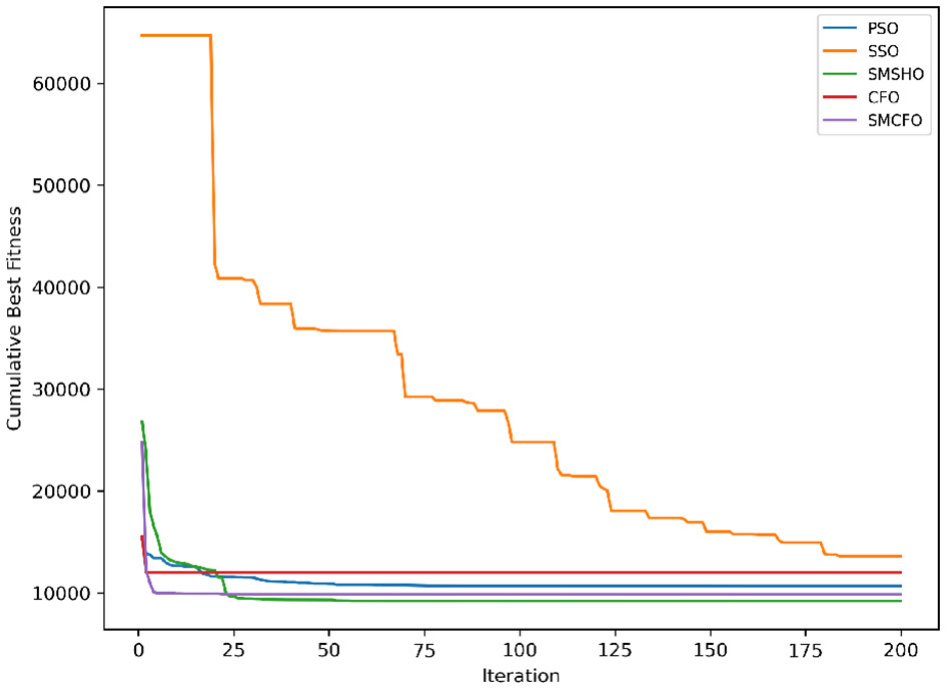
Evolutionary convergence trends for cmc.

**Figure 12:**
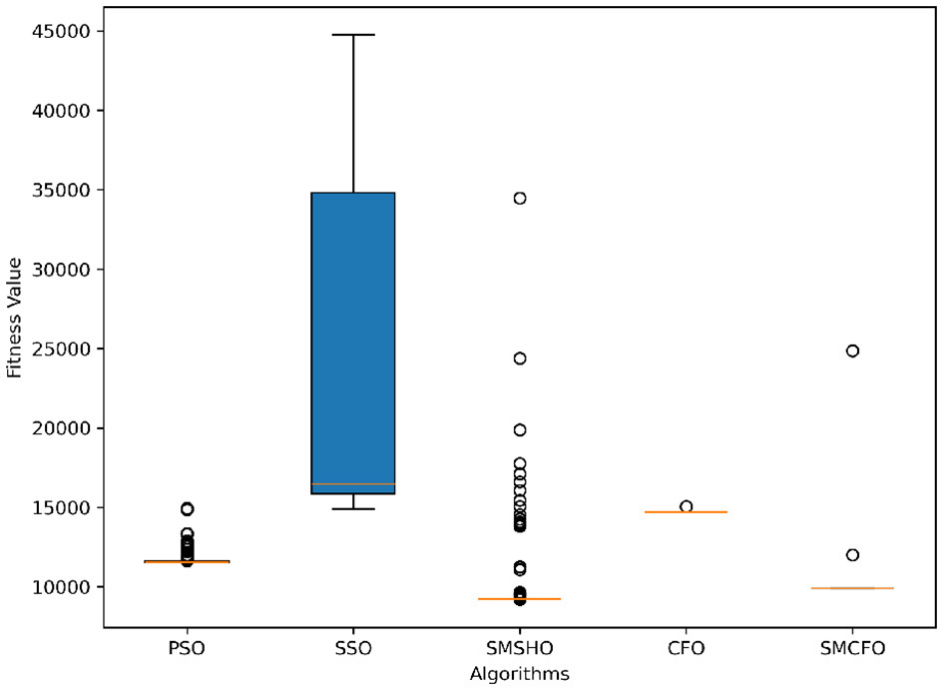
Variance analysis of optimization methods on cmc.

**Figure 13:**
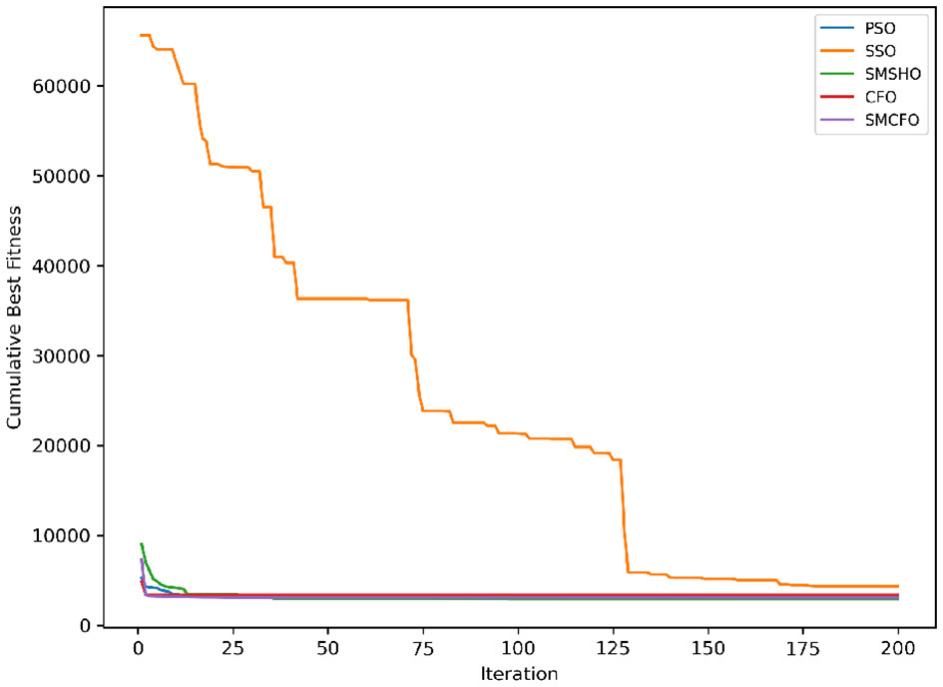
Evolutionary convergence trends for heartstatlog.

**Figure 14:**
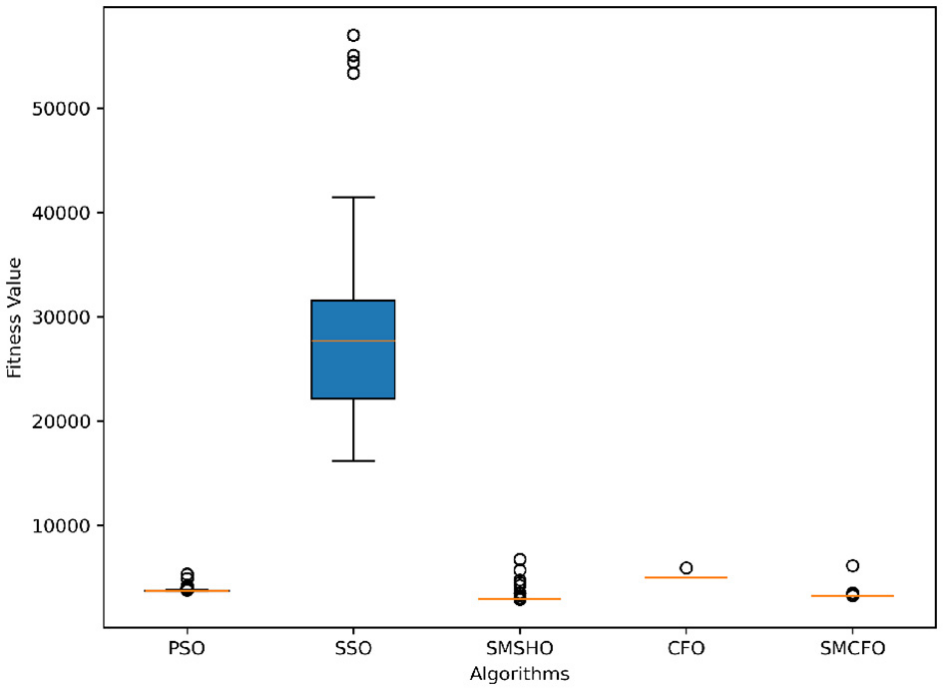
Variance analysis of optimization methods on heartstatlog.

**Figure 15:**
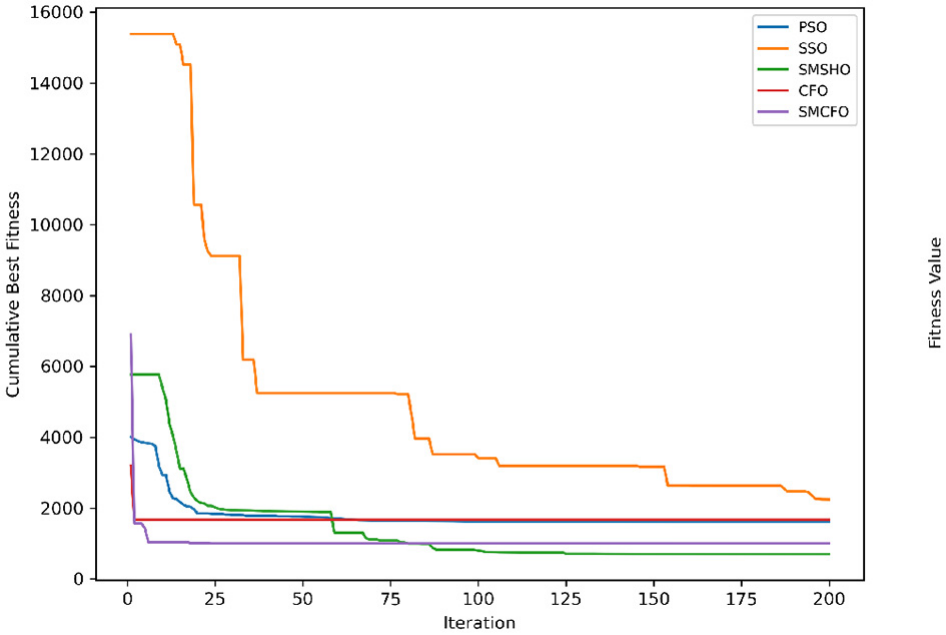
Evolutionary convergence trends for glass.

**Figure 16:**
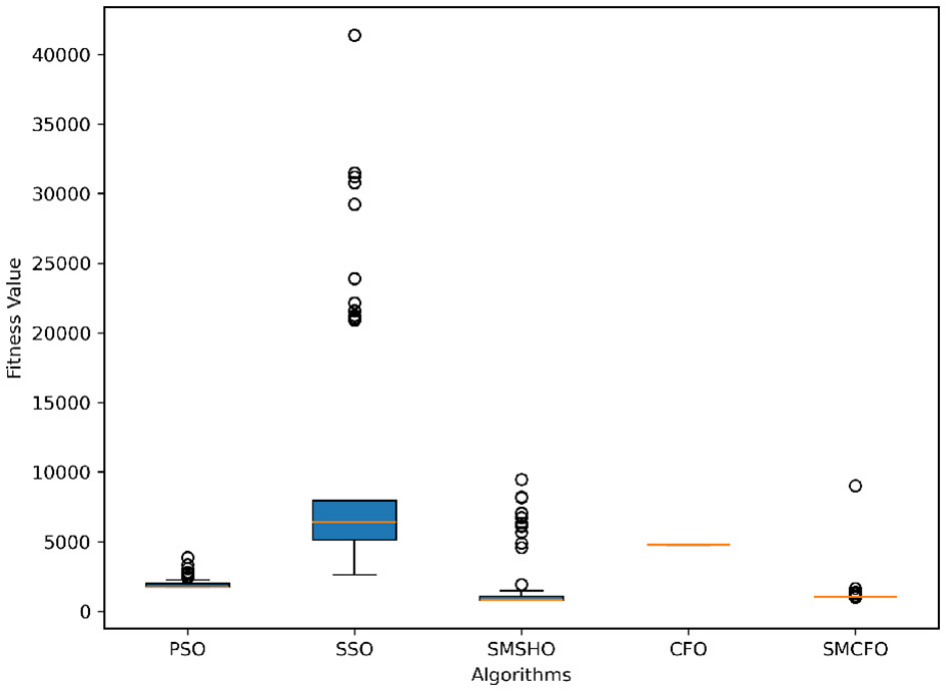
Variance analysis of optimization methods on glass.

**Figure 17:**
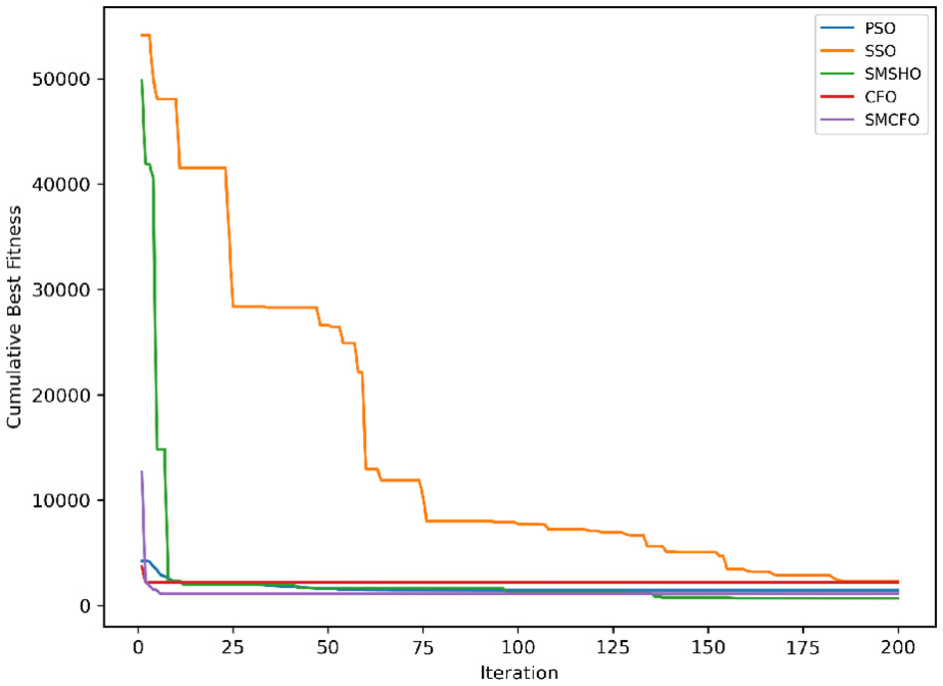
Evolutionary convergence trends for ecoli.

**Figure 18:**
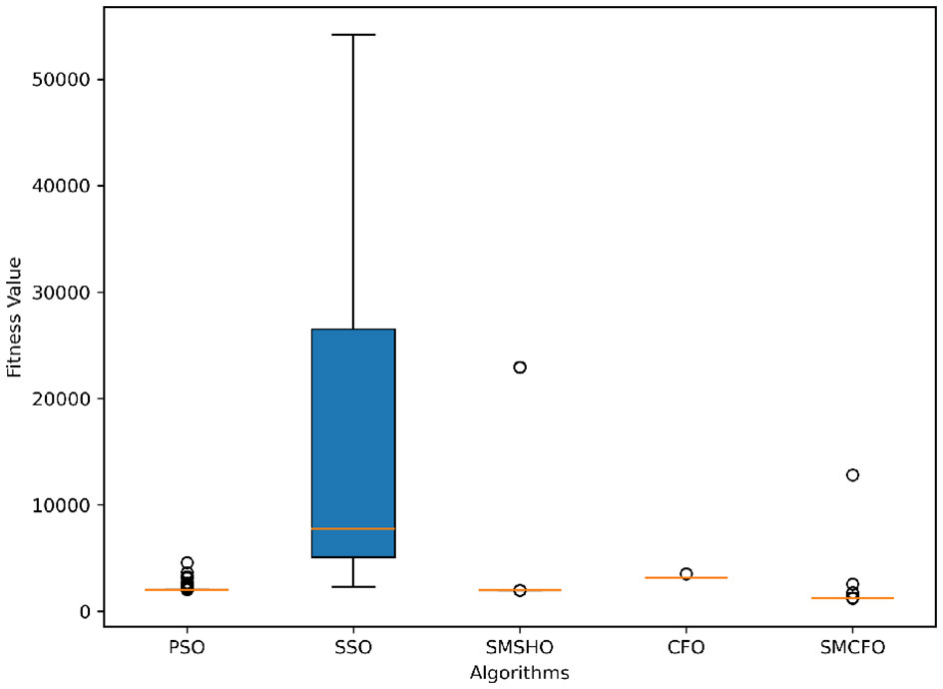
Variance analysis of optimization methods on ecoli.

The results summarized in Tables 2–15 demonstrate its competitive effectiveness in clustering optimization. Notably, SMCFO secured the highest ranking (1st place) on the iris, Breast Cancer, and vehicle datasets, indicating its superior capability. On the haberman, vertebral, and sonar datasets, SMCFO ranked 3rd, while in all remaining datasets, it consistently achieved 2nd place, showcasing its reliability and robustness across a diverse range of clustering tasks. A detailed comparison reveals that in instances where SMCFO ranked 2nd or 3rd, the top-performing algorithm was either SMSHO or PSO, suggesting that these two methods serve as its primary competitors. Despite these variations in ranking, SMCFO consistently outperformed CFO across all datasets, highlighting its overall advantage in terms of clustering accuracy and optimization efficiency. These findings reinforce the suitability of SMCFO as a competitive alternative to CFO, particularly in scenarios where enhanced clustering performance is required. As is evident from Figures 3–18, the proposed SMCFO algorithm achieves the most rapid convergence among all the other optimization methods, with the highest values for variance as well. The high variance indicates that the algorithm has extreme variations when performing optimization. Nevertheless, the variance is minimal for all datasets, which shows that the suggested algorithm generates consistent and stable results with fewer variations across varied datasets. This stability indicates that the algorithm is strong, with consistent performance even under divergent situations. Even with these changes, the overall performance of the proposed SMCFO method is still better than the other algorithms, showing that it is effective and reliable for solving the given clustering problem.

**Table 15:** Comparative analysis of algorithms on ecoli.

**Algorithm**	**Best**	**Worst**	**Mean**	**SD**	**Rank**
PSO	23.3869	37.6375	30.8817	0.7407	3
SSO	45.4966	958.6798	165.2995	107.1570	5
SMSHO	**13.8480**	**16.4804**	**14.5757**	**0.6861**	**1**
CFO	47.7837	188.0031	95.5133	22.8017	4
SMCFO	16.9548	69.5823	17.2179	3.7213	2

Bold values indicate the best performance achieved for each metric among all compared methods.

### 5.4 Clustering performance of the algorithm

The clustering procedure of the proposed SMCFO algorithm is analyzed, and its efficacy is visually evaluated using three datasets: art2, iris, and vehicle.

#### 5.4.1 Cluster process of SMCFO

**Figure 19:**
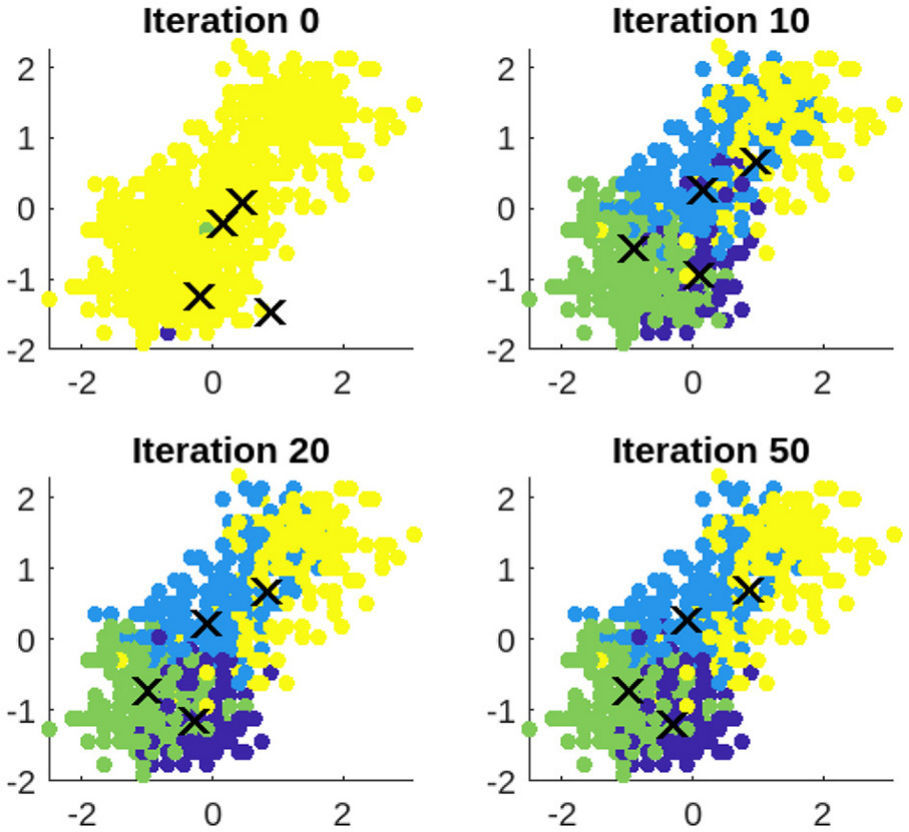
Clustering results of SMCFO for vehicle dataset over the iterations 0, 10, 20, 50.

The vehicle dataset is utilized to showcase the clustering process of SMCFO. Clustering results are presented at iterations 0, 10, 20, and 50 to provide a detailed visualization of the algorithm's progression. The experimental findings, illustrated in Figure 19, depict each cluster using a distinct color for clarity. As the number of iterations increases from 0 to 10, 20, and 50, the clustering of data points gradually improves. Initially, at iteration 0, the clusters may be poorly defined, with significant overlap and scattered points. By iteration 10, the clustering shows noticeable refinement, with data points beginning to align more closely with their respective clusters. At iteration 20, the cluster separation becomes more distinct, reducing noise and misclassified points. By iteration 50, the clustering reaches a more optimized state, with well-formed groups and minimal misclassification, indicating convergence toward a stable clustering solution.

### 5.5 Analyzing population diversity improvement in the SMCFO algorithm

A comparative analysis is conducted to demonstrate how the proposed SMCFO algorithm improves population diversity relative to the original algorithm.

#### 5.5.1 Mathematical formulation of population diversity

Optimization algorithms analyze how individuals disperse throughout the solution space to quantify population diversity. A more extensive distribution indicates greater diversity, which helps the algorithm explore more effectively and avoid getting stuck in local optima. Let the population *S* contain *N* individuals with fitness values *f*_1_, *f*_2_, …, *f*_*N*_. Calculate the mean fitness of the population using


(20) $$\begin{array}{l} \bar{f}=\frac{1}{N}\overset{N}{\underset{i=1}{\sum }}f_{i} \end{array}$$

The Spatial Dispersion Index (SDI) is defined as


(21) $$\begin{array}{l} \text{SDI}=\frac{\sum_{i=1}^{N}{\left(f_{i}-\bar{f}\right)}^{2}}{N\cdot {\bar{f}}^{2}} \end{array}$$

This expression describes the variation of the fitness values across the individuals of the population. Because every individual's fitness reflects their location in the search space, an increase in SDI indicates greater spatial dispersal of the individuals, hence more diversity. A low SDI indicates the population resides within a small, dense search space region, which indicates convergence. So, the SDI is a significant parameter to consider while studying an algorithm's diversity dynamics under optimization.

#### 5.5.2 Analysis of population diversity

**Figure 20:**
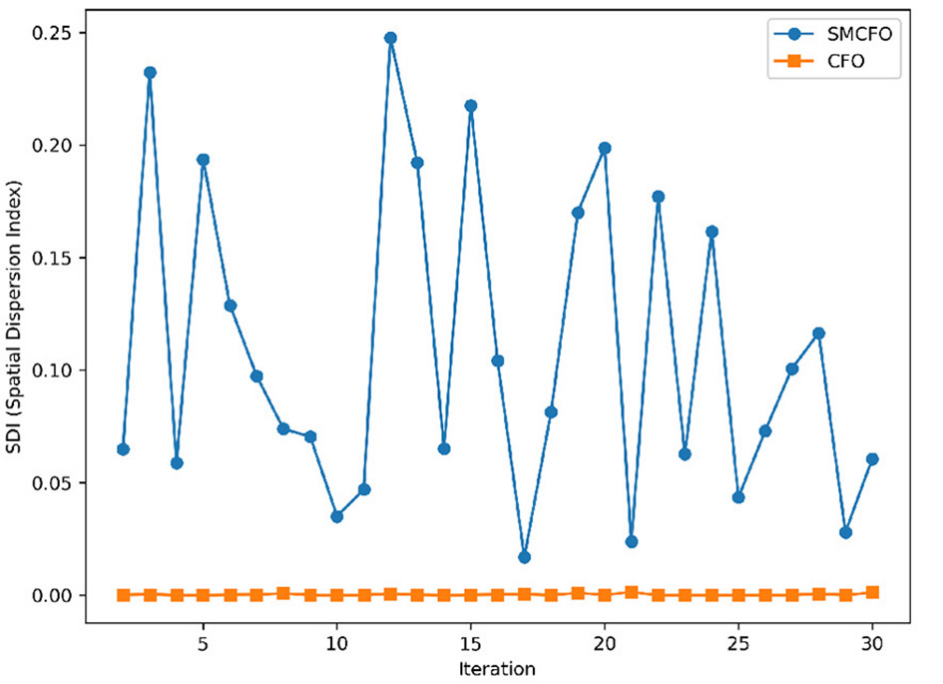
Variance distribution analysis in art1.

**Figure 21:**
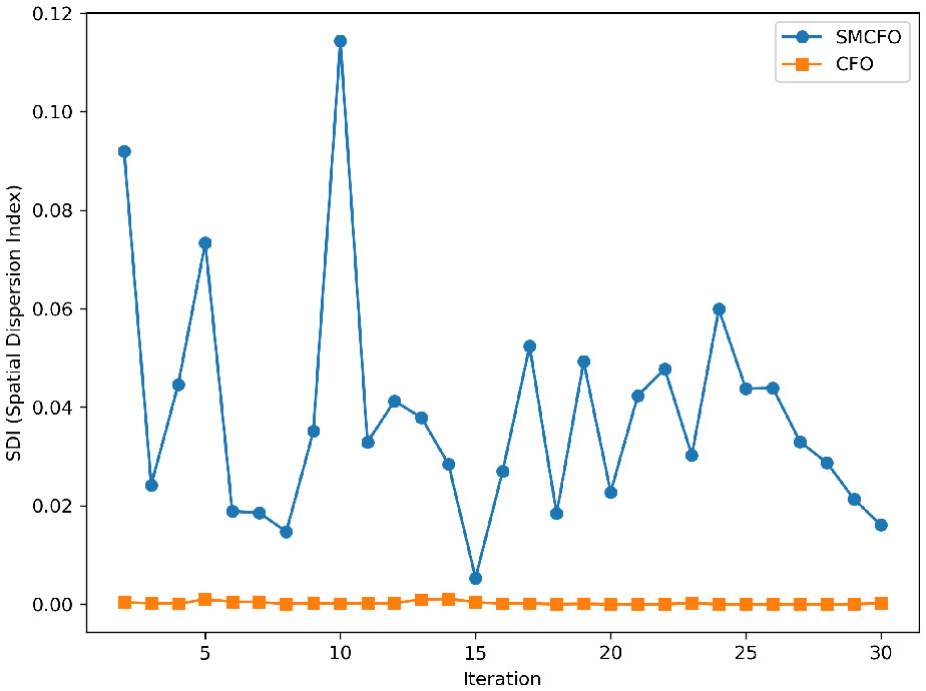
Variance distribution analysis in art2.

**Figure 22:**
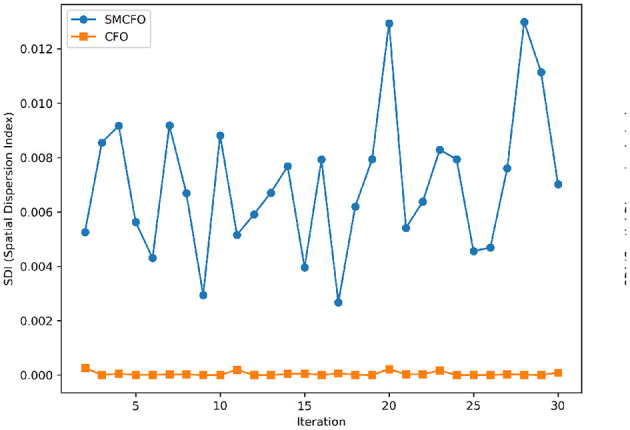
Variance distribution analysis in tae.

**Figure 23:**
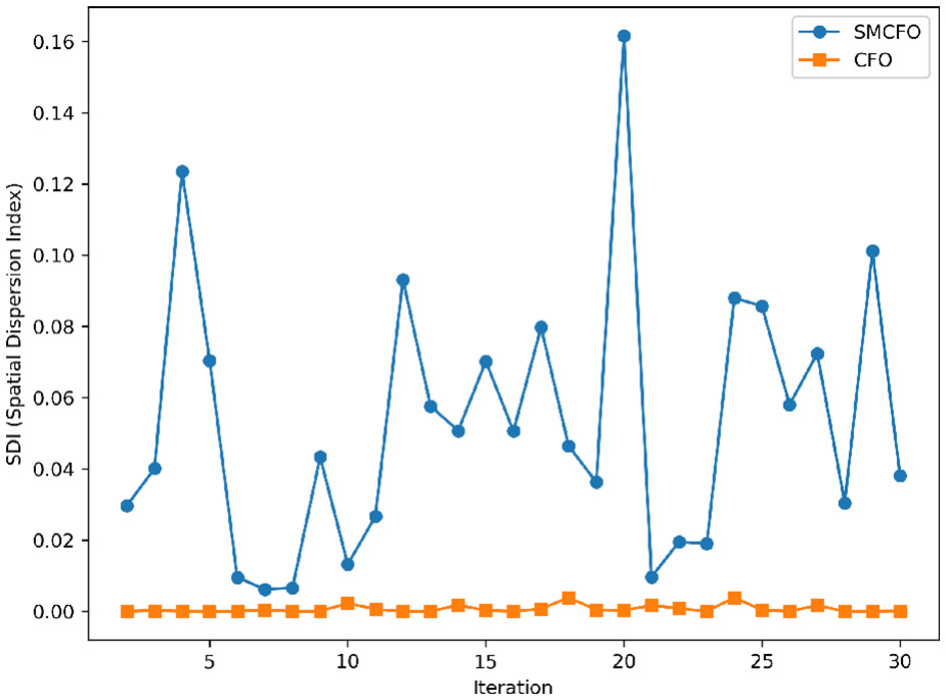
Variance distribution analysis in thy.

**Figure 24:**
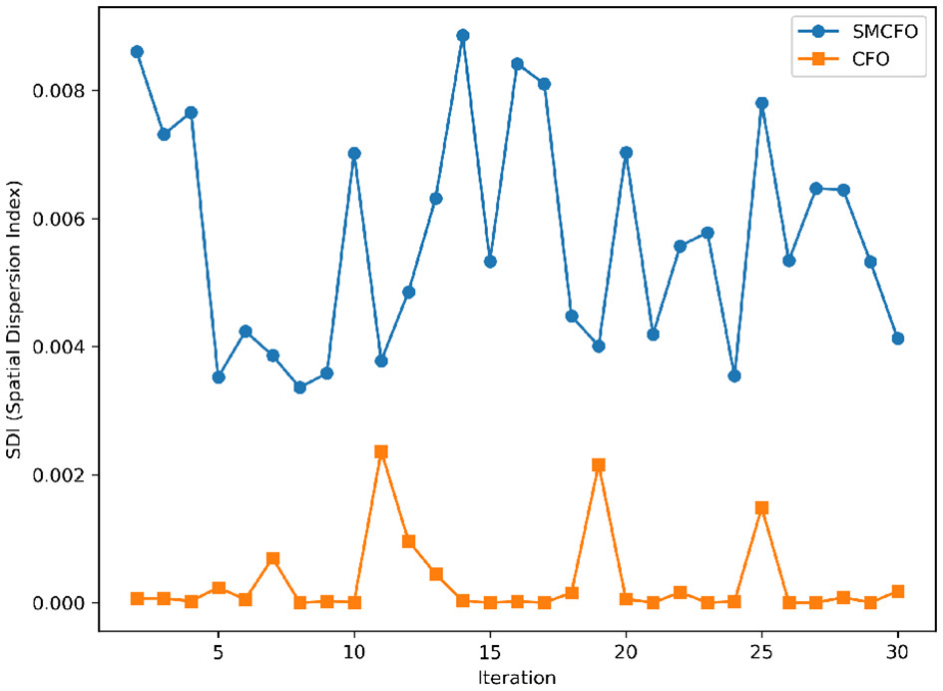
Variance distribution analysis in heartstatlog.

**Figure 25:**
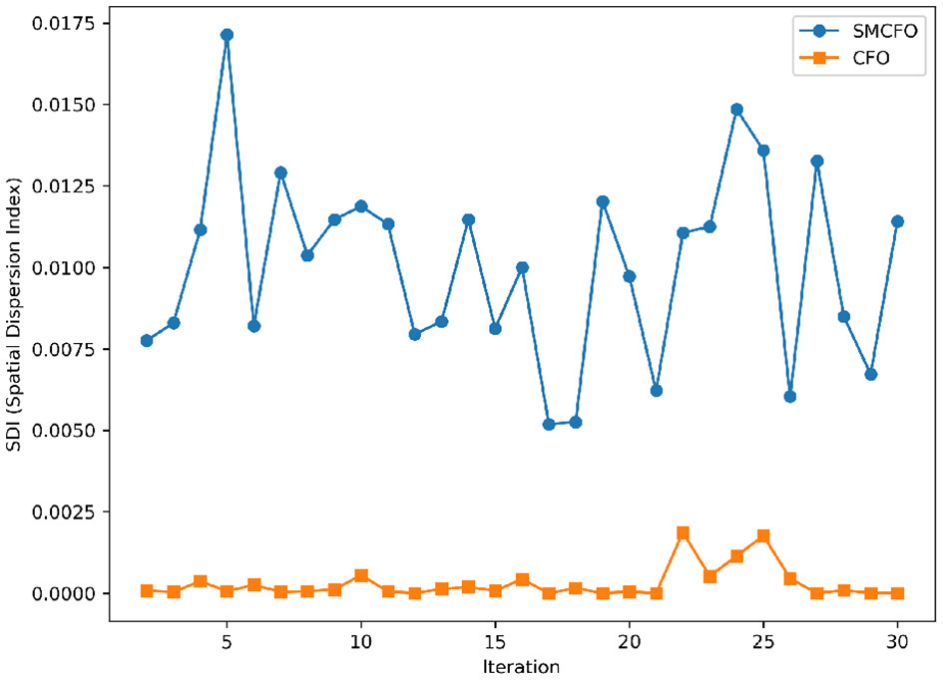
Variance distribution analysis in cmc.

**Figure 26:**
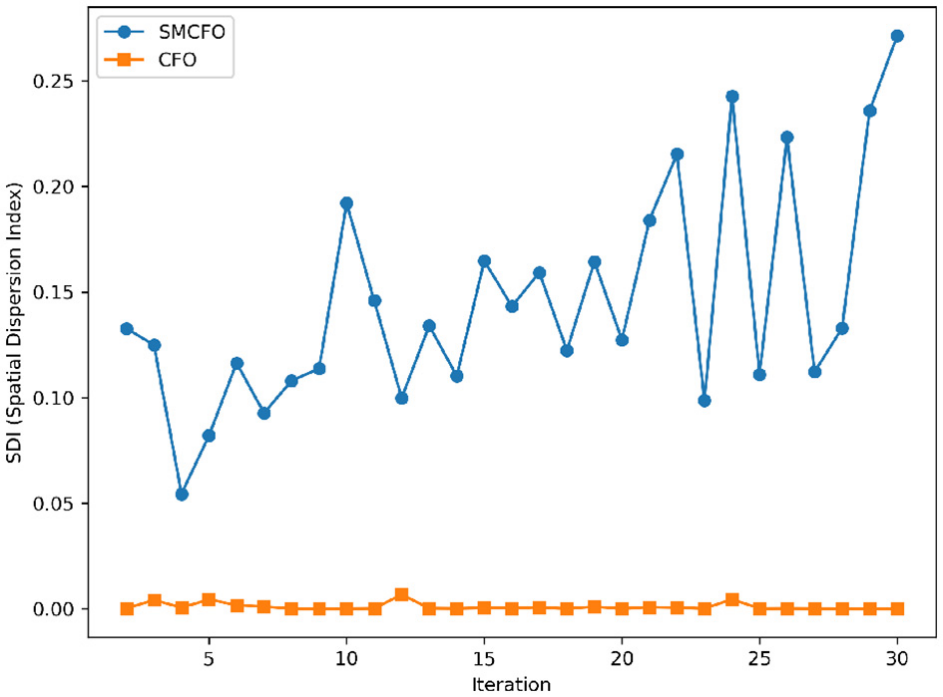
Variance distribution analysis in glass.

**Figure 27:**
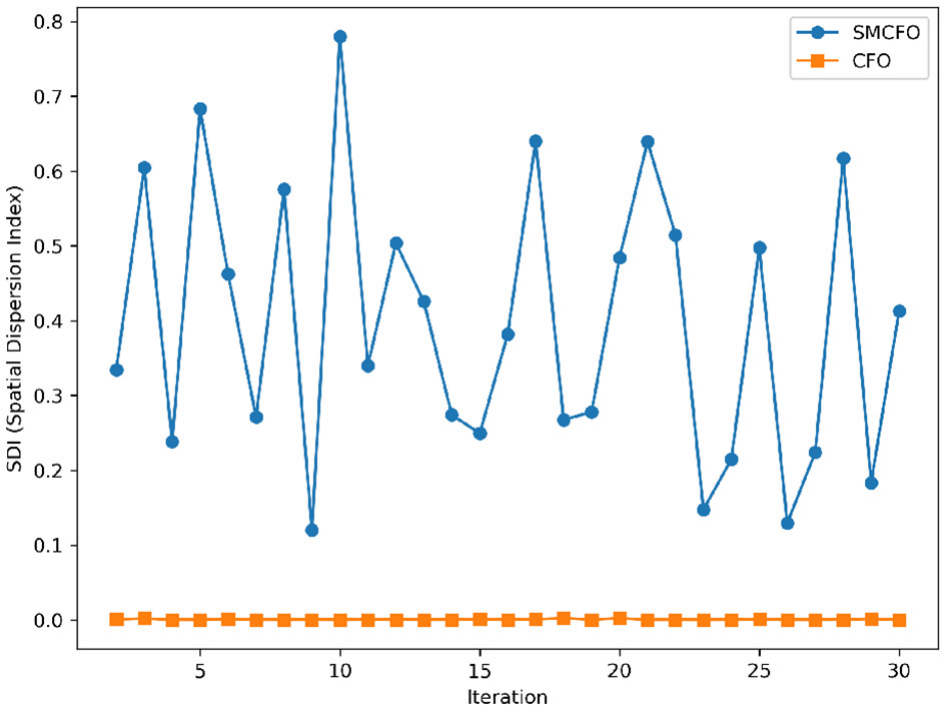
Variance distribution analysis in ecoli.

The algorithm illustrates greater population diversity during the earlier phases of the search process. Higher spatial distribution of individuals improves the chances of reaching the global optimum. The fitness values continue to converge to the global optimum as the search advances, which causes a reduction in population diversity. The study highlights the importance of early search iterations to enable a meaningful comparative assessment. Variance values are analyzed at the 15^th^, 20^th^, 25^th^, and 30^th^ iterations. Table 16 presents experimental results, and Figures 20–27 present variance trends for the art1, art2, tae, thy, cmc, heartstatlog, glass, and ecoli datasets. Table 16 illustrates that the new algorithm always generates a higher variance of fitness values than the existing algorithm in early iterations. The distribution of broader solutions indicates greater population diversity and better global search ability. Figures 20–27 demonstrate that the suggested approach has more variance than the original algorithm during the initial optimization phase. This high variance is not a random fluctuation. Still, it follows a stable and consistent pattern, proving that the proposed algorithm's increased population diversity is systematic and not random.

**Table 16:** Analysis of population diversity.

**Database**	**15th**		**20th**		**25th**		**30th**	
	**SMCFO**	**CFO**	**SMCFO**	**CFO**	**SMCFO**	**CFO**	**SMCFO**	**CFO**
art1	**2.32E-01**	1.30E-03	**2.04E-01**	1.00E-03	**1.80E-01**	8.36E-04	**1.60E-01**	7.03E-04
art2	**3.24E-01**	7.40E-03	**3.09E-01**	5.70E-03	**2.87E-01**	4.60E-03	**2.70E-01**	3.90E-03
tae	**4.57E-02**	8.00E-04	**4.04E-02**	6.13E-04	**3.56E-02**	4.97E-04	**3.16E-02**	4.18E-04
thy	**2.00E-01**	3.41E-04	**1.68E-01**	2.61E-04	**1.44E-01**	2.11E-04	**1.26E-01**	1.77E-04
cmc	**5.85E-02**	0.00E+00	**4.87E-02**	0.00E+00	**4.15E-02**	0.00E+00	**3.61E-02**	0.00E+00
heartstatlog	**4.32E-02**	2.28E-06	**3.45E-02**	1.74E-06	**2.87E-02**	1.41E-06	**2.45E-02**	1.18E-06
glass	**8.05E-01**	1.11E-04	**7.11E-01**	8.45E-05	**6.31E-01**	6.84E-05	**5.65E-01**	5.74E-05
ecoli	**1.89E+00**	9.44E-04	**1.82E+00**	7.24E-04	**1.73E+00**	5.87E-04	**1.66E+00**	4.93E-04

Bold values indicate the best performance achieved for each metric among all compared methods.

### 5.6 Stability evaluation of the proposed SMCFO algorithm

**Figure 28:**
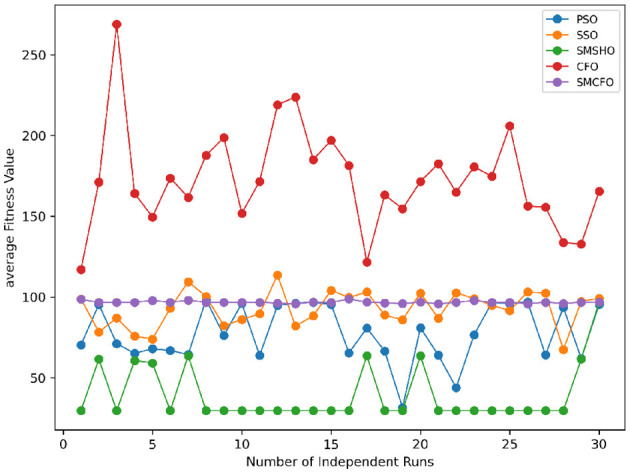
Convergence stability visualization for art1.

**Figure 29:**
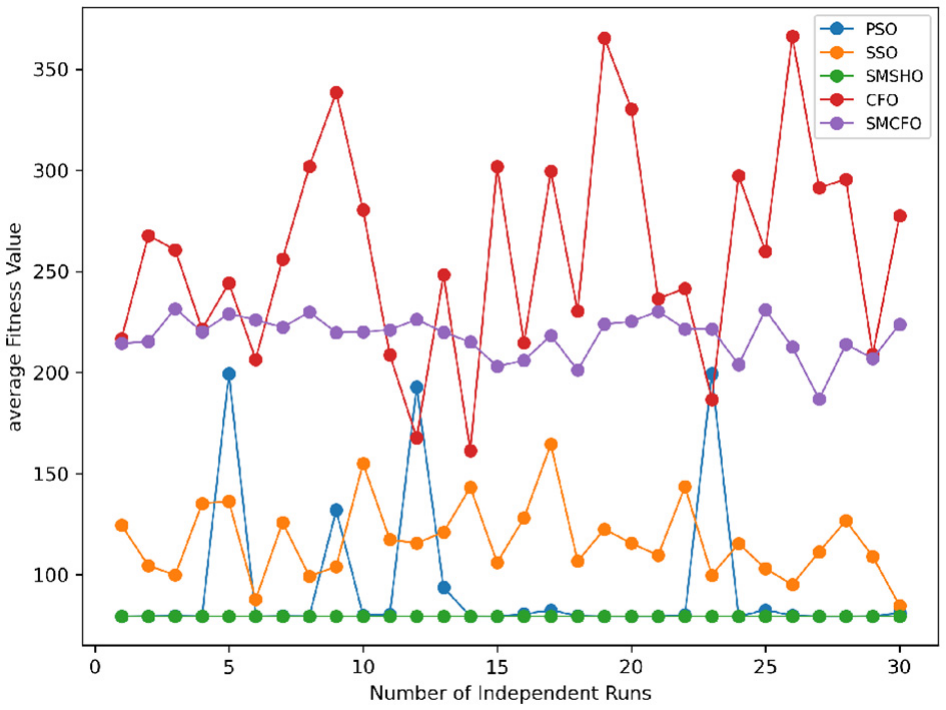
Convergence stability visualization for art2.

**Figure 30:**
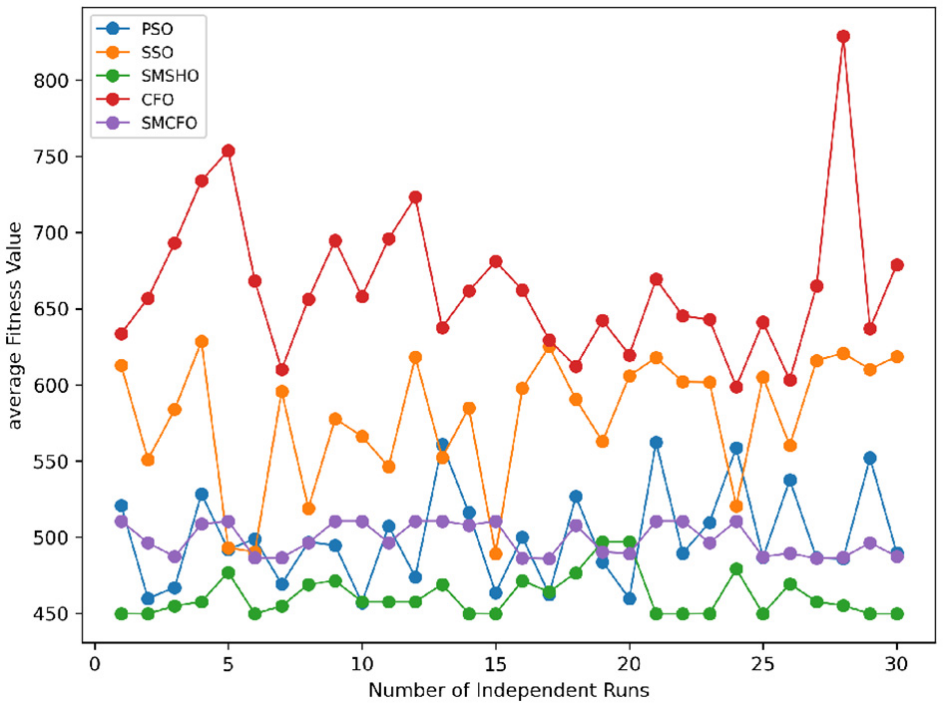
Convergence stability visualization for tae.

**Figure 31:**
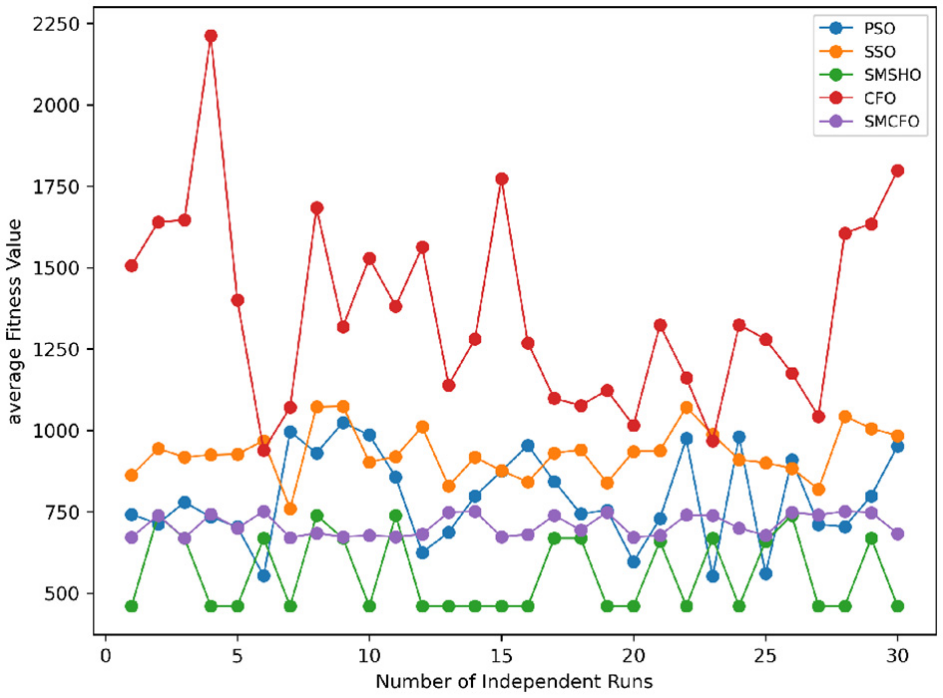
Convergence stability visualization for thy.

**Figure 32:**
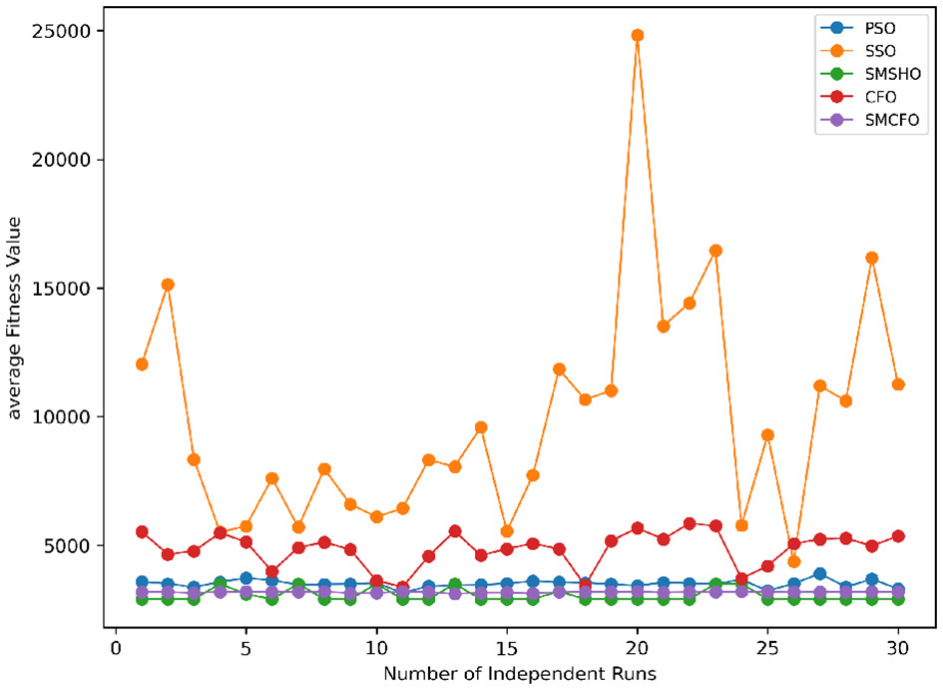
Convergence stability visualization for heartstatlog.

**Figure 33:**
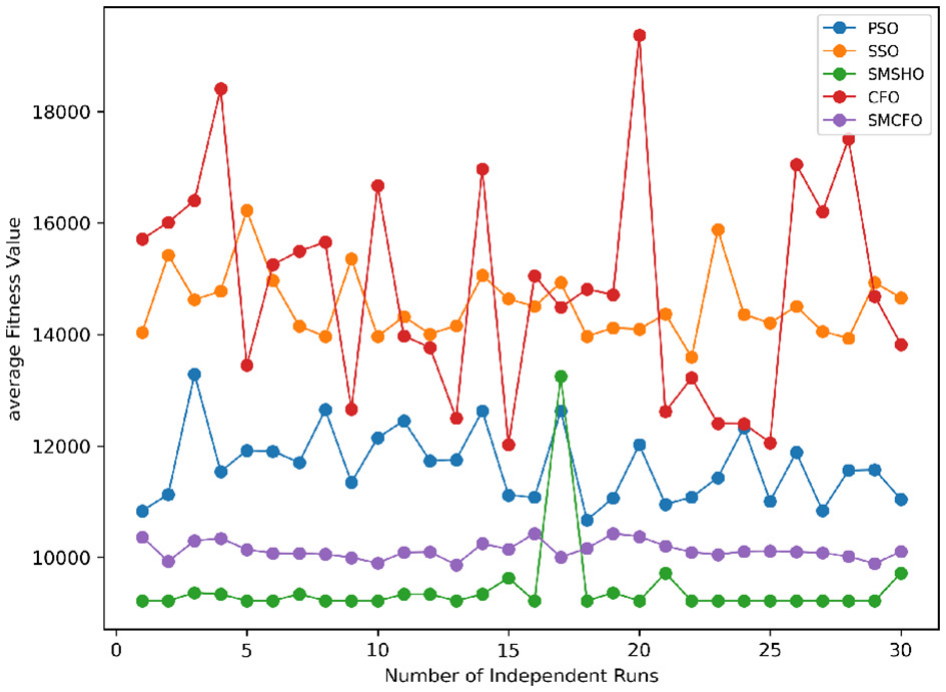
Convergence stability visualization for cmc.

**Figure 34:**
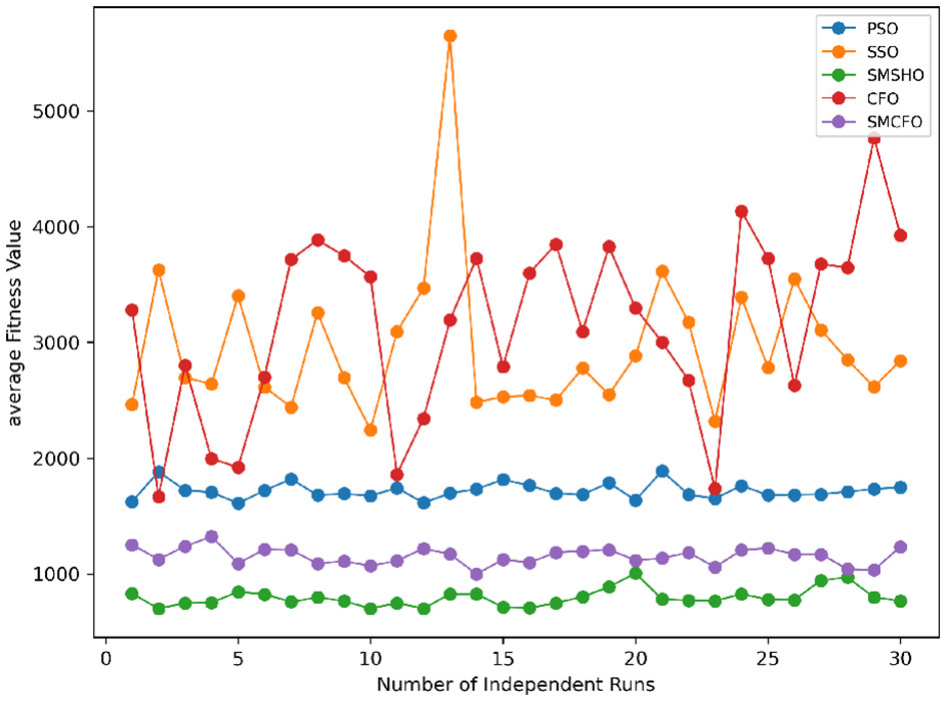
Convergence stability visualization for glass.

**Figure 35:**
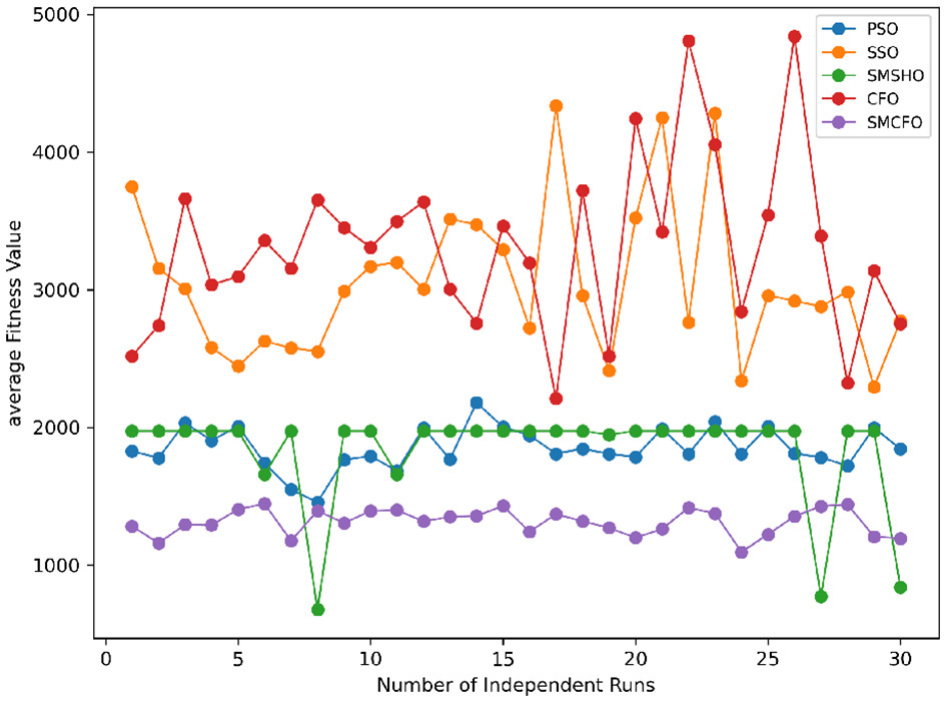
Convergence stability visualization for ecoli.

Stability of the proposed algorithm is evaluated using experimental data from 30 independent runs, as detailed in Section 5.3. The assessment includes all considered algorithms—PSO, SSO, SMPSO, CFO, and SMCFO—across benchmark datasets. Figures 28–35 present the stability curves of all the evaluated algorithms–PSO, SSO, SMPSO, CFO, and the proposed SMCFO–across various benchmark datasets. The stability plots measure the distribution of the obtained fitness values based on 30 independent runs of each algorithm. Across the datasets, the proposed algorithm shows minimal or zero variance in its fitness values at all points, unlike the original algorithms, which show higher variability. Such stability of performance guarantees that the proposed approach achieves a more stable and uniform convergence process. The minor variance demonstrates the strength of the proposed algorithm. It confirms its ability to maintain solution quality under repeated runs, thereby constructing its superior stability compared to the baseline algorithms.

### 5.7 Runtime efficiency analysis of the algorithms

The runtime comparison between SMCFO and CFO across benchmark datasets highlights the efficiency gains and the computational overhead introduced by the simplex-enhanced design. SMCFO provides significant performance in datasets such as art1, art2, thy, cmc, and heartstatlog, as Table 17 shows. art2 shows the most significant improvement, with a 70% reduction in execution time from 45.25 to 13.33 seconds. These cases highlight how the Nelder-Mead simplex method accelerates convergence and reduces computational cost when the search space is relatively low-dimensional or moderately complex.

**Table 17:** Runtime comparison between CFO and SMCFO (in seconds).

**Dataset**	**CFO (s)**	**SMCFO (s)**		
		**Group 1**	**Other groups**	**Total**
art1	26.9499	4.367446	0.002073	4.602394
art2	45.2508	3.166557	0.002456	3.525148
tae	8.2126	3.716139	0.002738	3.935072
thy	11.7591	3.360865	0.002441	3.576417
heartstatlog	10.4010	5.268119	0.002396	5.539633
cmc	78.8871	23.112601	0.002922	24.035071
glass	27.8973	28.813956	0.003201	29.240945
ecoli	48.5128	79.383515	0.003828	80.456814

In contrast, on datasets such as glass and ecoli, the runtime of SMCFO exceeds that of CFO. Simplex-based refinement, which typically requires several objective assessments every iteration in higher-dimensional or noisy feature spaces, is the cause of this increase. In addition, cluster size also matters: the more clusters there are, the more expensive centroid updates and simplex refinements become, resulting in increased runtime overhead.

These parameters may enhance the clustering quality even though they lengthen runtimes by creating better centroids and stable partitions. Even though SMCFO is more computationally expensive in complex scenarios because runtime efficiency is not the top priority, the balance favors effectiveness. The balance between accuracy, stability, and speed must be essential in clustering tasks.

### 5.8 Impact of population size on the performance of the algorithm

The proposed SMCFO algorithm demonstrates enhanced performance through a comprehensive evaluation across multiple benchmark datasets and population sizes. SMCFO consistently achieves lower or comparable mean fitness values to the original CFO, particularly at smaller populations such as 20, 40, and 60. Table 18 shows that SMCFO can explore the solution space more effectively and converge toward optimal solutions faster, even with fewer search agents. For instance, at a population size of 40, the fitness value for the art2 dataset dropped from 1,198 (CFO) to 252.62 (SMCFO), showcasing an impressive improvement in solution quality. In addition to solution quality, SMCFO demonstrates improved stability and robustness across diverse datasets with varying feature sizes and class distributions. Unlike CFO, which often shows flat performance regardless of population variation, SMCFO adapts dynamically and produces competitive results even on complex datasets such as thy, glass, and ecoli. While in some cases, such as at higher population sizes (e.g., 80), SMCFO incurs slightly higher fitness values due to increased complexity in local search computations, the overall trend favors the proposed method regarding both effectiveness and efficiency. SMCFO's ability to balance global exploration with local exploitation more precisely makes it a superior choice for clustering tasks where both accuracy and computational cost are critical.

**Table 18:** Mean fitness comparison of CFO and SMCFO at different population sizes.

**Dataset**	**Pop** = **20**		**Pop** = **40**		**Pop** = **60**		**Pop** = **80**	
	**CFO**	**SMCFO**	**CFO**	**SMCFO**	**CFO**	**SMCFO**	**CFO**	**SMCFO**
art1	747	163.32	747	96.69	747	96.69	315.53	1,747.49
art2	1,198	2,653.88	1,198	252.62	1,198	252.62	353.39	3,275.76
tae	750	546.84	750	522.28	750	518.18	1,298.78	1,521.39
thy	1,070	851.81	1,070	699.55	1,070	699.55	8,381.63	8,109.11
heartstatlog	3,497	3,307.17	3,497	3,166.31	3,497	3,166.31	9,900.78	7,441.88
cmc	13,248	9,996.73	13,248	9,906.84	13,248	99,06.84	35,728.93	50,485.71
glass	1,917	1,437.23	1,917	1,219.43	1,917	1,219.43	10,087.00	18,417.50
ecoli	2,345	3,916.32	2,345	1,270.59	2,345	1,270.59	6,508.79	27,062.64

### 5.9 Effect of iteration count on algorithmic performance

As evident from Tables 19, 20, the findings highlight the performance of CFO and SMCFO algorithms at various iterations with all parameters fixed as per Section 5.3, with the only variable being the number of iterations. These findings reveal that SMCFO always performs better than CFO in terms of stability, speed of convergence, and overall dependability. While SMCFO maintains steady fitness values with minimal fluctuations across iterations, CFO exhibits significant variations, particularly in the later stages. This trend is most apparent in complex datasets like ecoli and glass, where SMCFO converges more efficiently and avoids the more significant deviations observed in CFO. In addition, SMCFO is more stable and performs steadily across iterations, whereas CFO performs slower and with more considerable variation. SMCFO's stability indicates its better capability to optimize solutions and escape local minima, making it a more consistent option for clustering operations on varied datasets. These findings highlight the benefits of incorporating the Nelder-Mead simplex method within Cuttlefish Optimization so that SMCFO can realize accelerated, more regular, and more stable convergence and thus outperform the original CFO algorithm.

**Table 19:** Mean fitness values for CFO across different iterations.

**Dataset**	**Iteration**				
	**50**	**100**	**150**	**250**	**300**
art1	683.73	683.73	683.73	683.73	683.73
art2	489.46	1,084.33	1,084.34	1,084.34	1,084.34
tae	843.90	856.62	856.62	856.62	856.62
thy	2,432.36	2,432.36	2,432.36	2,432.36	2,432.36
heartstatlog	5,289.49	5,313.35	5,313.35	5,313.35	5,313.35
cmc	16,206.37	17,338.48	17,346.32	17,346.32	17,346.32
glass	3,472.99	3,472.99	3,472.99	3,472.99	3,472.99
ecoli	4,833.70	5,137.75	4,848.18	4,848.18	4,848.18

**Table 20:** Mean fitness values for SMCFO across different iterations.

**Dataset**	**Iteration**				
	**50**	**100**	**150**	**250**	**300**
art1	469.96	450.22	450.22	450.22	450.22
art2	1,044.50	1,014.18	1,066.41	1,013.06	1,064.79
tae	875.56	877.16	869.29	863.00	863.06
thy	3,439.07	3,439.10	3,439.07	3,439.10	3,439.07
heartstatlog	5,667.50	5,670.91	5,664.09	5,676.94	5,665.23
cmc	20,097.47	20,055.23	20,130.73	20,055.34	20,130.62
glass	5,382.42	5,374.69	5,374.88	5,374.83	5,374.51
ecoli	12,591.03	12,585.61	12,584.86	12,596.35	12,605.38

### 5.10 Statistical validation using nonparametric tests

The Wilcoxon rank-sum test (Derrac et al., 2011), a widely used nonparametric statistical method, was used to strictly test the statistical significance of differences in performance between algorithms. The test is suitable for comparing two independent samples when the data does not follow a normal distribution. It tests whether one group tends to have higher values than another without assuming the underlying distribution, making it best suited for testing algorithm performance on diverse datasets.

The statistical inference was based on the mean values reported in Tables 2–15. A p-value was calculated for every pairwise comparison between the proposed SMCFO approach and the four baseline methods: CFO, PSO, SSO, and SMSHO. The p-value quantifies the probability of achieving the observed outcome under the null hypothesis that there is no statistically significant difference between the mean performance of the two algorithms. A smaller p-value provides more substantial evidence against the null hypothesis.

The study used a significance level of 5%(α = 0.05), a standard cut-off value in statistical hypothesis testing. This cutoff was chosen because it strikes an equilibrium between reducing false positives (Type I error) and avoiding actual effects from being missed (Type II error). This represents a 5% chance of incorrectly concluding the presence of a difference when no difference exists. A stricter 1% significance level (α = 0.01) could lower the likelihood of Type I errors but might increase the risk of Type II errors, leading to the possible failure to detect significant differences. Accordingly, the 5% level provides a reasonable compromise, allowing rigorous but not unduly inhibiting testing.

Table 21 lists the p-values obtained using the Wilcoxon rank-sum test. In most comparisons, the p-values are well below 0.05, providing strong statistical evidence against the null hypothesis.

**Table 21:** Wilcoxon rank-sum test p-values for sMCFO vs. other algorithms.

**Dataset**	**SMCFO vs**.			
	**CFO**	**PSO**	**SSO**	**SMSHO**
art1	1.0386E-80	2.8903E-72	7.1408E-68	1.5468E-75
art2	5.9107E-75	7.6675E-71	1.9002E-25	2.4159E-81
tae	4.0974E-77	2.4371E-71	2.3473E-68	2.6633E-65
thy	3.1709E-80	2.1449E-71	2.1689E-70	9.6101E-48
heartstatlog	1.0693E-84	1.3498E-74	6.4545E-74	2.8339E-77
cmc	6.1420E-81	7.0467E-72	5.9534E-53	8.2430E-74
glass	7.4065E-85	7.1716E-75	6.5139E-75	2.6391E-06
ecoli	2.92E-82	6.71E-73	5.80E-72	3.34E-12

Table 22 shows the Friedman test findings confirm statistically significant differences between the compared algorithms (*p* < 0.05 across all datasets). In particular, SMCFO routinely places in the top two performers on every dataset. SMCFO frequently competes with PSO or SMSHO and is explicitly ranked as the best or second-best in several datasets ( art1, tae, thy, hearstatlog, glass, ecoli). Table 23 shows the Nemenyi *post-hoc* test in that SMCFO achieves statistically significant improvements over CFO, PSO, and SSO in most datasets, with very small p-values (e.g., 10^−9^, 10^−12^, 10^−14^). Against SMSHO, SMCFO shows mixed results: in some cases, differences are not significant (glass, *p* = 0.4292), while in others SMCFO outperforms SMSHO (e.g., ecoli, *p* = 0.0037).

**Table 22:** Friedman test results with best rankings.

**Dataset**	**SMCFO**	**CFO**	**PSO**	**SSO**	**SMSHO**	**Friedman_p**	**First_Best**	**Second_Best**
art1	1.995	3.465	5	3.510	1.030	7.0637e-161	SMSHO	SMCFO
art2	2.770	3.875	5	2.355	1.000	1.4063e-158	SMSHO	SSO
tae	2.005	3.210	5	3.775	1.010	8.0466e-165	SMSHO	SMCFO
thy	1.905	3.660	5	3.330	1.105	2.6036e-160	SMSHO	SMCFO
heartstatlog	3.005	3.995	1	5	2.000	1.1423e-171	PSO	SMSHO
cmc	2.075	3.260	1	4.060	4.605	5.5555e-148	PSO	SMCFO
glass	2.475	3.925	1	5	2.600	1.4647e-159	PSO	SMCFO
ecoli	2.290	3.955	1	5	2.755	9.1675e-163	PSO	SMCFO

**Table 23:** Nemenyi *post-hoc* test *p*-values for SMCFO vs. other algorithms.

**Dataset**	**SMCFO vs**.			
	**CFO**	**PSO**	**SSO**	**SMSHO**
art1	0	0	0	1.0397E-09
art2	2.7758E-12	0	0.0086728	0
tae	2.5091E-14	0	0	3.1152E-10
thy	0	0	0	4.2004E-07
heartstatlog	3.8176E-10	0	0	2.0684E-10
cmc	6.6391E-14	1.0542E-11	0	0
glass	0	0	0	0.4292
ecoli	0	4.4409E-16	0	0.0032724

The findings show that the performance gains achieved by the proposed SMCFO algorithm are due to its effective design, not random variation. Therefore, the SMCFO algorithm significantly and consistently outperforms the other algorithms on all tested datasets.

### 5.11 Clustering quality comparison: SMCFO vs. CFO

The proposed SMCFO algorithm is compared with the original CFO approach using eight benchmark datasets to evaluate clustering performance. The evaluation relied on standard clustering performance metrics, including accuracy, F-measure, sensitivity (detection rate), specificity, and adjusted Rand index (ARI). Table 24 shows that the SMCFO algorithm consistently outperformed CFO across most datasets, particularly in accuracy, where it achieved higher values on seven out of eight datasets, such as 0.7317 on art2 compared to CFO's 0.5. SMCFO continually enhanced precision-recall trade-offs and better identified positive cluster assignments, especially in datasets such as art1 and heartstatlog, echoing the same trends in F-measure and sensitivity. Though both algorithms retained high specificity, SMCFO performed better on datasets such as glass and ecoli, efficiently eliminating false positives. In terms of clustering agreement, ARI scores were consistently higher with SMCFO, indicating greater alignment with ground truth labels–for example, art1 (0.8481 vs. 0.8269), art2 (0.8345 vs. 0.6437), and ecoli (0.7362 vs. 0.4658). These results demonstrate that the modifications introduced in SMCFO enhance clustering quality, stability, and convergence, particularly in datasets characterized by complexity or imbalance.

**Table 24:** Clustering quality comparison: SMCFO vs. CFO.

**Dataset**	**SMCFO**					**CFO**				
	**Acc**.	**F-meas**.	**Sens**.	**Spec**.	**ARI**	**Acc**.	**F-meas**.	**Sens**.	**Spec**.	**ARI**
art1	0.712	0.636	0.712	0.928	0.848	0.600	0.490	0.600	0.900	0.827
art2	0.732	0.637	0.732	0.911	0.834	0.500	0.334	0.500	0.833	0.644
tae	0.377	0.249	0.378	0.689	0.386	0.430	0.395	0.436	0.718	0.509
thy	0.730	0.405	0.411	0.703	0.580	0.716	0.344	0.371	0.694	0.569
heartstatlog	0.611	0.610	0.623	0.623	0.523	0.641	0.550	0.598	0.598	0.538
cmc	0.427	0.199	0.333	0.667	0.353	0.482	0.342	0.399	0.708	0.487
glass	0.355	0.105	0.168	0.834	0.297	0.411	0.215	0.285	0.861	0.534
ecoli	0.640	0.395	0.416	0.935	0.736	0.488	0.221	0.237	0.893	0.466

### 5.12 Limitations and future directions

While SMCFO provides better stability and centroid accuracy on average, it is not always better. In datasets with greater dimensionality or strict evaluation budgets, the simplex step incurs per-iteration overhead that lowers the rate of global updates, at times constraining exploration. The local refinement can over-exploit shallow basins in datasets with overlapping classes and noisy or redundant features (e.g., glass). Class skewness and numerous small clusters (e.g., ecoli) also prevent centroid placement with a strictly Euclidean objective. For these, we see more minor improvements or some underperformance compared to baselines. To achieve this, future updates will (i) adaptively trigger simplex based on recent progress and use it only for top candidates, (ii) limit per-iteration simplex evaluations to safeguard the global search budget, (iii) include dimensionality reduction or feature weighting/metric learning to help counteract noise and overlap, (iv) introduce diversity-preserving restarts or annealed exploration weights, and (v) leverage parallelization of simplex evaluations. These modifications preserve SMCFO's advantages in accuracy while enhancing its robustness on complex datasets.

## 6 Conclusion

The effectiveness of the proposed SMCFO method is assessed using benchmark datasets representing diverse tasks such as species classification, disease prediction, educational assessment, analysis of contraceptive behavior, signal processing, and protein localization. The SMCFO algorithm integrates the Nelder-Mead Simplex method into the original CFO framework, replacing the traditional reflection and visibility stages for group 1. The primary motivation for introducing the simplex method is to enhance the algorithm's exploration capability and population diversity. This combination allows for creating a more extensive and diverse range of candidate solutions in the search space, which enhances the likelihood of obtaining improved fitness values and strengthens the capacity of the algorithm to escape local optima. The SMCFO algorithm improves global search ability and demonstrates better convergence speed and stability. In data clustering problems, every solution in SMCFO is a collection of cluster centroids. The performance of the proposed algorithm is tested using extensive experiments on two artificial and twelve real-world datasets. Its performance is compared with well-known algorithms, such as PSO, SSO, SMSHO, and CFO. Both numerical and graphical results affirm that SMCFO outperforms the other methods consistently, with improved fitness values, stable convergence behavior, and high capability for avoiding premature convergence.

Future research will concentrate on a few essential topics. Firstly, will look into complex fitness function designs to improve performance on noisy and unbalanced datasets. Further illustrating the practical utility of SMCFO, will thoroughly assess its scalability and dependability on large-scale, multi-modal, high-dimensional datasets. In addition, SMCFO will expand to address complex real-world optimization issues, such as intelligent transportation systems, blockchain transaction data for scalable and fraud-proof clustering, image segmentation, and scheduling jobs.

## Data Availability

The original contributions presented in the study are included in the article/supplementary material, further inquiries can be directed to the corresponding author.
